# Network failures: When incentives trigger impulsive responses

**DOI:** 10.1002/hbm.24941

**Published:** 2020-03-09

**Authors:** Peter Zhukovsky, Sharon Morein‐Zamir, Chun Meng, Jeffrey W. Dalley, Karen D. Ersche

**Affiliations:** ^1^ Behavioural and Clinical Neuroscience Institute, University of Cambridge Cambridge UK; ^2^ Department of Psychology University of Cambridge Cambridge UK; ^3^ School of Psychology and Sports Science Anglia Ruskin University Cambridge UK; ^4^ Department of Psychiatry University of Cambridge Cambridge UK

**Keywords:** fMRI, impulsivity, monetary incentive delay, stimulant drug dependence

## Abstract

Adequate control of impulsive urges to act is demanded in everyday life but is impaired in neuropsychiatric conditions such as stimulant use disorder. Despite intensive research it remains unclear whether failures in impulse control are caused by impaired suppression of behavior or by the over invigoration of behavior by stimuli associated with salient incentives such as drugs, food, and money. We investigated failures in impulse control using functional magnetic resonance imaging (fMRI) to map the neural correlates of premature (impulsive) responses during the anticipation phase of the Monetary Incentive Delay (MID) task in healthy controls (HC), stimulant‐dependent individuals (SDIs), and their unaffected first‐degree siblings (SIB). We combined task‐based fMRI analyses with dynamic causal modeling to show that failures of impulse control were associated with interactions between cingulo‐opercular and dorsal striatal networks regardless of group status and incentive type. We further report that group‐specific incentive salience plays a critical role in modulating impulsivity in SDIs since drug‐related incentives specifically increased premature responding and shifted task modulation away from the dorsal striatal network to the cingulo‐opercular network. Our findings thus indicate that impulsive actions are elicited by salient personally‐relevant incentive stimuli and those such slips of action recruit a distinct fronto‐striatal network.

## 1 INTRODUCTION

Behavior is often driven by the motivational properties of rewards, yet the interactions between motivation and cognitive control and their neural substrates are unclear (Botvinick & Braver, 2015; Hull, 1943). In the Principles of Psychology, William James stated that motivated behaviors “are but results of the fact that certain things appeal to primitive and instinctive impulses of our nature, and that we follow their destinies with an excitement that owes nothing to a reflective source” (James, 1890), suggesting that motivated behavior can be accompanied by weakened “reflective” cognitive control over one's impulses.

Impulsivity has been linked to numerous neuropsychiatric disorders and, as a multifaceted construct, incorporates a range of different traits and behaviors (Dalley, Everitt, & Robbins, 2011; Evenden, 1999). Key aspects of impulsivity include risky decision making and impulsive action (Dalley & Robbins, 2017). Impulsive action is typically gauged by assessing the tendency to act prematurely without foresight or sufficient regard to negative consequences, or by assessing the ability to countermand or stop a prepotent response (Robbins et al., 2012). Evidence from humans and rodents suggests the two complementary forms of impulsive action rely on overlapping, yet distinct neural networks and neurochemical substrates (Dalley & Robbins, 2017). While stopping prepotent responses is associated with dorsal striatal (dStriatum) along with ventrolateral and dorsomedial prefrontal (PFC) involvement (Swick et al., 2011), premature responding appears to involve the ventral striatum and ventromedial PFC (Dalley et al., 2011).

The inability to suppress inappropriate responses is believed to play a key role in stimulant drug addiction both as a vulnerability factor and as a consequence of chronic drug use (Goldstein & Volkow, 2011). This can manifest in the day‐to‐day lives of drug users as difficulties in suppressing excessive approach behaviors and urges to act, particularly when exposed to incentivizing drug‐related cues. Converging evidence shows that chronic stimulant drug use is associated with response inhibition impairments and top‐down cognitive control abnormalities underpinned by aberrant fronto‐striatal function (Morein‐Zamir & Robbins, 2015). Unaffected siblings of stimulant dependent individuals (SDI) also show response inhibition difficulties and associated structural brain abnormalities, suggesting this may be a preexisting vulnerability factor (Ersche et al., 2012a). While decisional impulsivity is often investigated in incentivized contexts (Kjome et al., 2010; Vigil‐Colet, 2007), impulsive actions in humans have largely been investigated by assessing stopping or canceling prepotent responses in non‐incentivized contexts (Bari & Robbins, 2013).

Findings on the neural correlates of premature responding in incentivized contexts have largely stemmed from rodent research (Dalley et al., 2008; Eagle & Baunez, 2010). Here, premature responses gauge difficulties in suppressing responses by capturing the inability to resist responding until a waiting interval has elapsed. Premature responding predicts the transition to compulsive cocaine‐taking in rodents, suggesting it could also be a vulnerability factor (Belin et al., 2008). Recent studies using a specialized paradigm adapted from the animal literature have begun to make inroads, demonstrating increased premature responding in abstinent SDIs (Voon, 2014). Greater premature responding has also been reported for tobacco smokers, cannabis users, and binge drinkers (Mechelmans et al., 2017; Morris et al., 2016; Sanchez‐Roige et al., 2014; Voon et al., 2016) reinforcing the importance of this measure to addiction more broadly.

As noted above, impulsive actions are clearly intertwined with reward processing. Empirical research into mechanisms of reward has proceeded largely in parallel. This research has pointed to aberrant generalized reward processing in addiction, largely as a consequence of drug use (Balodis & Potenza, 2015; Cope et al., 2019; Koob & Moal, 2005). For example, mesocortico‐limbic abnormalities are believed to underscore exaggerated incentive salience of drug‐associated stimuli (Berridge, 2007). Thus, the process of “wanting” triggered when faced with drug‐related cues yields upregulation of reward‐related regions (Berridge, 2007). In humans, the widely‐used monetary incentive delay (MID) task has been employed to assess reward related processing in addiction (Luijten et al., 2017). Anticipating monetary rewards in this task has been associated with robust activation in ventral striatum in addition to dorsal striatum and vlPFC activations (Oldham et al., 2018). Contrary to expectations, drug users including SDIs do not appear to show consistent abnormalities in cross‐sectional MID studies (Balodis and Potenza, 2015; Just et al., 2019). One possible reason for this may be the use of monetary incentives in these studies. Addiction is associated with blunted brain response in a wide array of non‐drug‐related tasks, but with increased engagement of brain networks during exposure to drug cues or drug‐related incentives (Zilverstand, Huang, Alia‐Klein, & Goldstein, 2018). This is underscored in the theory of impaired response inhibition and salience attribution (iRISA) which points to the pivotal role of context and incentive type (Goldstein & Volkow, 2002; Goldstein & Volkow, 2011).

The current study investigates the neural correlates of premature responses in stimulant users and their unaffected siblings and healthy controls in different incentive conditions using functional Magnetic Resonance Imaging (fMRI). We focus on premature responding in the MID task (Peña‐Oliver et al., 2016), introducing a novel analysis approach. Premature responses are akin to the everyday maladaptive behaviors exhibited by these individuals and to our knowledge their neural underpinnings using fMRI have not yet been directly explored. By including two distinct contexts, one providing monetary cues as incentives and the other drug‐related cues as incentives, we provide a direct empirical test of the iRISA model predictions. We predicted drug related cues to elicit increased impulsive behavior and increased activation in fronto‐striatal networks. Assessing the SIB allowed us to investigate whether excessive premature responding and corresponding brain activations constitutes a vulnerability factor preceding drug use (Just et al., 2019). Given the importance of the striatum to top‐down control and reward processing in addiction and its abnormal neuronal connectivity with the PFC (Ma et al., 2014; Ma et al., 2015), we also investigated the selective involvement of this region and its effective connectivity using dynamic causal modeling (DCM) within key nodes of the PFC.

## 2 METHODS

### 2.1 Participants

Participants were recruited for this study by advertisements, by word of mouth, and from local treatment services (see Table 1 for demographic information). Recruitment and screening procedures have been described in detail elsewhere (Ersche et al., 2012b; Just et al., 2019). Briefly, three groups consisted of SDIs who met Diagnostic Statistical Manual (DSM‐IV‐TR) criteria for cocaine or amphetamine dependence, their biological siblings who had no history of substance dependence except nicotine, and healthy individuals without familial risk with no drug history. Urine screen results were positive for all but three SDI and negative for all other participants. The study was approved by the NHS Cambridge Research Ethics Committee (08/H0308/310) and all participants provided written informed consent. Data from these individuals as part of a larger sample have been published previously (Just et al., 2019). This study introduces novel approach to evaluate premature responses in the MID task taking advantage of the incentivized setting. Additional inclusion criteria here were that participants exhibit at least one premature response in the MID task in each context.

**Table 1:** Demographic, personality, and clinical measures for the three groups. Data are means ± *SD*. Significant differences (*p* < .05) are highlighted in bold; Chi‐square tests were used for categorical comparisons and one‐way analyses of variance were used to test continuous outcomes. Gender (Chi^2^ = 36.48, *p* < .001), Impulsivity (Barratt Impulsivity questionnaire, BIS‐11, *F*_2,123_ = 38.22, *p* < .001), and value money (“How likely are you to pick up 10p/50p?”, *F*_2,123_ = 4.439, *p* = .0138) were significantly different between the HC, SIB, and SDI groups

	Healthy controls (*n* = 42)		At‐risk siblings (*n* = 43)		Stimulant dependent individuals(*n* = 41)	
Demographics	Mean	(*SD*)	Mean	(*SD*)	Mean	(*SD*)
Age (years)	32.6	(8.8)	32.3	(8.4)	34.7	(7.4)
Gender (% male)	64.3		47.8		88.1	
Verbal intelligence (NART)	112.0	(8.4)			110.6	(7.4)
Monthly disposable income (£)	695	1,000	403	410	399	672
Duration of stimulant use (years)					16.1	(6.5)
Compulsive stimulant use (OCDUS)					23.6	(9.3)
Impulsivity (BIS‐11)	59.7	(7.9)	67.3	(10.5)	77.3	(9.3)
Value money ratings	71.3	(27.0)	58.6	(32.8)	76.1	(26.8)

Abbreviations: BIS‐11, Barret Impulsivity Scale; NART, National Adult Reading Test; OCDUS, Obsessive Compulsive Drug Use Scale.

### 2.2 MID task

**Figure 1:**
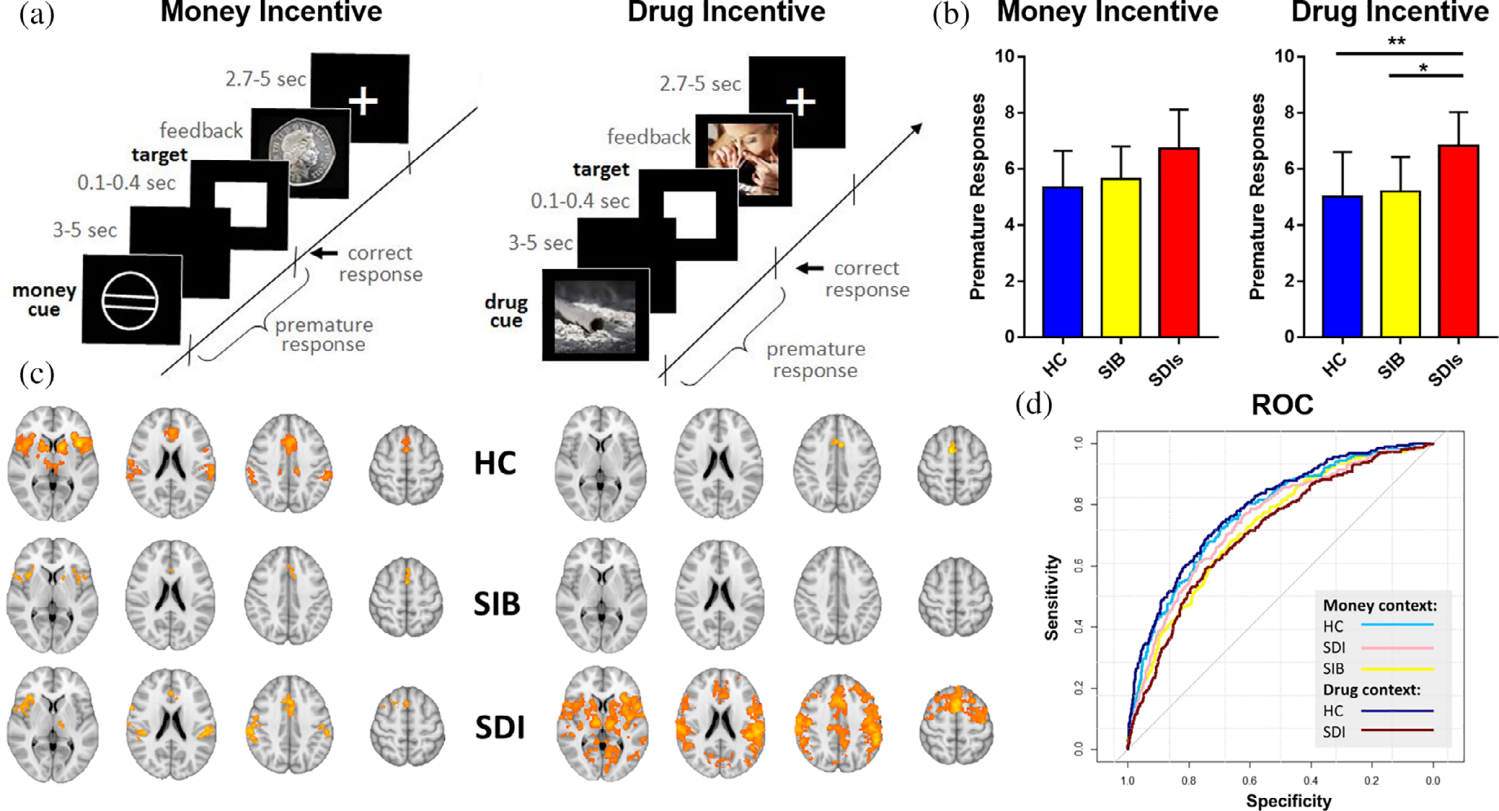
Impulsive responding when anticipating an incentive. (a) Cues indicated the incentive available on each trial, and participants prepared during the anticipation phase to respond during the brief period when the target was presented. Incentives could be monetary (a small sum of money) or drug‐related images, with 44 incentive and 22 neutral trials in each of the two contexts. (b) The number of premature responses, where participants responded during the anticipation phase and before the target appeared, for each of the three groups and to the two incentives. Post‐hoc tests with false discovery rate correction were used. (c) Axial brain slices (*z* = 4, 21, 39, 56) demonstrating prefrontal and striatal activations when participants responded prematurely during the anticipation phase (whole brain cluster corrected *z* = 2.3, *p* = .05). (d) Receiver Operating Curves demonstrating good model performance of regions of interest timeseries in predicting whether participants responded prematurely or accurately to the target. Asterisks in figure denote level of significance (*p < .05; **p < .01)

The task consisted of money and drug incentive blocks, with the two counterbalanced across subjects. While the stimuli displayed differed between the two contexts, timings and task structure were the same. Each incentive block consisted of 66 trials, of which 22 were neutral. Trials began with a cue (lasting 250 ms) signaling the reward. For money incentives, a circle with two, one or no horizontal lines indicated a possible win of 50 pence (large reward), 10 pence (small reward) or 0 pence (neutral), respectively. For drug incentives, images of cocaine, crack, IV or non‐IV drugs (white powder) in commonly taken form, or a bottle of water as neutral, were used to signal the expected reward (see Figure 1a). Following cue presentation, participants awaited a target cue for an anticipation period lasting 3,000–5,000 ms. Subsequently, a white square target was presented, lasting 100–400 ms, with participants required to press a key while the target was still on screen. Target duration was titrated to maintain a 66% success rate. Target presentation was followed by a feedback message (1,650 ms). For money incentives, successful responding was followed by a message “you've won 10p/50p” along with the respective coin. Neutral and unsuccessful trials were followed by a message “you've won 0 p” accompanied by a white circle. Participants received monetary rewards conditional on their performance in the monetary block at the end of the task. For drug incentives, successful responding was followed by an image of a person taking cocaine, crack, IV or non‐IV drugs for the reward conditions, or a person drinking from a clear water bottle for the neutral or unsuccessful trials. An inter‐trial interval lasted 2,700–5,700 ms, during which a fixation cross was displayed. Participants first completed 66 practice trials prior to scanning with both incentive types.

### 2.3 Neuroimaging acquisition

Images were collected on a Siemens TIM Trio 3‐Tesla scanner (Erlangen, Germany) using whole‐brain echo planar images for functional data and T1 images for high resolution structural data. The following parameters were applied for the functional scan: repetition time = 2,000 ms; echo time = 30 ms, flip angle = 78°; 32 slices with a thickness of 3 mm plus a 0.75 mm gap; matrix = 64 × 64 field of view = 192 × 192 mm with an in‐plane resolution of 3 × 3 mm. T1 scans were acquired using: TR = 2,300 ms; TE = 2.98 ms; TI = 900 ms; flip angle = 9°; FOV = 240 × 256 mm, resulting in 176 slices of 1 mm thickness.

### 2.4 Data analysis

#### 2.4.1 Task performance

Analyses contrasted the number of premature responses between groups, for the money and drug contexts. As data were positively skewed and deviated significantly from the normal distribution (Kolmogorov–Smirnov tests, *p* < .05), one‐way nonparametric comparisons using Kruskal‐Wallis tests were conducted in each incentive condition and Dunn's post hoc tests were used for pairwise group comparisons.

#### 2.4.2 Imaging data

Following the discarding of the first 5 volumes, 330 volumes were analyzed in each incentive context. First level analyses were carried out using FSL FEAT (FMRI Expert Analysis Tool, http://www.fmrib.ox.ac.uk/fsl) with standard settings in FSL 6.0. Image preprocessing included brain extraction using BET (Smith, 2002) nonlinear registration of T1 images to Montreal Neurological Institute (MNI) standard space with 10 mm warps and boundary‐based registration of the functional image to the corresponding T1 image. Isotropic smoothing kernel with Gaussian full width half measure of 5 mm was chosen. Second level analyses were in MNI standard space (resampled to 2 × 2 × 2 mm). Motion correction (mcflirt, Jenkinson, Bannister, Brady, & Smith, 2002) was used to linearly register all images in a 4D volume to an average image and to estimate head motion parameters (rotation, temporal derivatives). Temporal derivatives of explanatory variables (EVs) were included in the first level General Linear Model (GLM), in lieu of slice‐timing correction.

These analyses focused on trials where participants responded during the anticipation window, that is, before target presentation. For the GLM fMRI analyses, premature responses were matched with corresponding correct trials where participants responded during target presentation. Specifically, a correct trial of the same type (e.g., neutral) appearing as close in presentation as possible to the premature trial was selected. Matching correct trials could occur both before and after the corresponding premature trial. This procedure ensured that contrasts included the same number of premature and correct events. For the purpose of the logistic regression fMRI analysis, all correct trials were used.

The first level design matrix included six EVs and their temporal derivatives: (a) premature responses for money incentive, (b) corresponding correct responses for money incentive, (c) premature responses with drug cue incentives, (d) corresponding correct responses for drug incentives, (e) all remaining correct responses, and (f) all feedback events. EV1–EV5 were modeled with event onset and duration as the start and duration of the anticipation window. EV6 was modeled with onset times and durations for the feedback. Effects of motion were controlled by including 24 motion parameters in the design matrix. Parameter estimate contrasts were calculated for EV1‐EV2 (premature > correct_money_) and for EV3‐EV4 (premature > correct_drug_).

Whole brain maps from the first level analyses were passed to a second level design matrix that tested mean group activations and pairwise group differences using one‐sample and independent sample *t*‐tests, respectively. Second level analyses used FEAT GLM with FLAME1 and a cluster forming threshold of *z* = 2.3 and voxel‐wise threshold of *p* < .05. Demeaned gender was included as a covariate in the GLM. The striatum region of interest (ROI) mask was created by combining bilateral masks for caudate, putamen, and nucleus accumbens from the Harvard‐Oxford atlas (Desikan et al., 2006).

**Figure 2:**
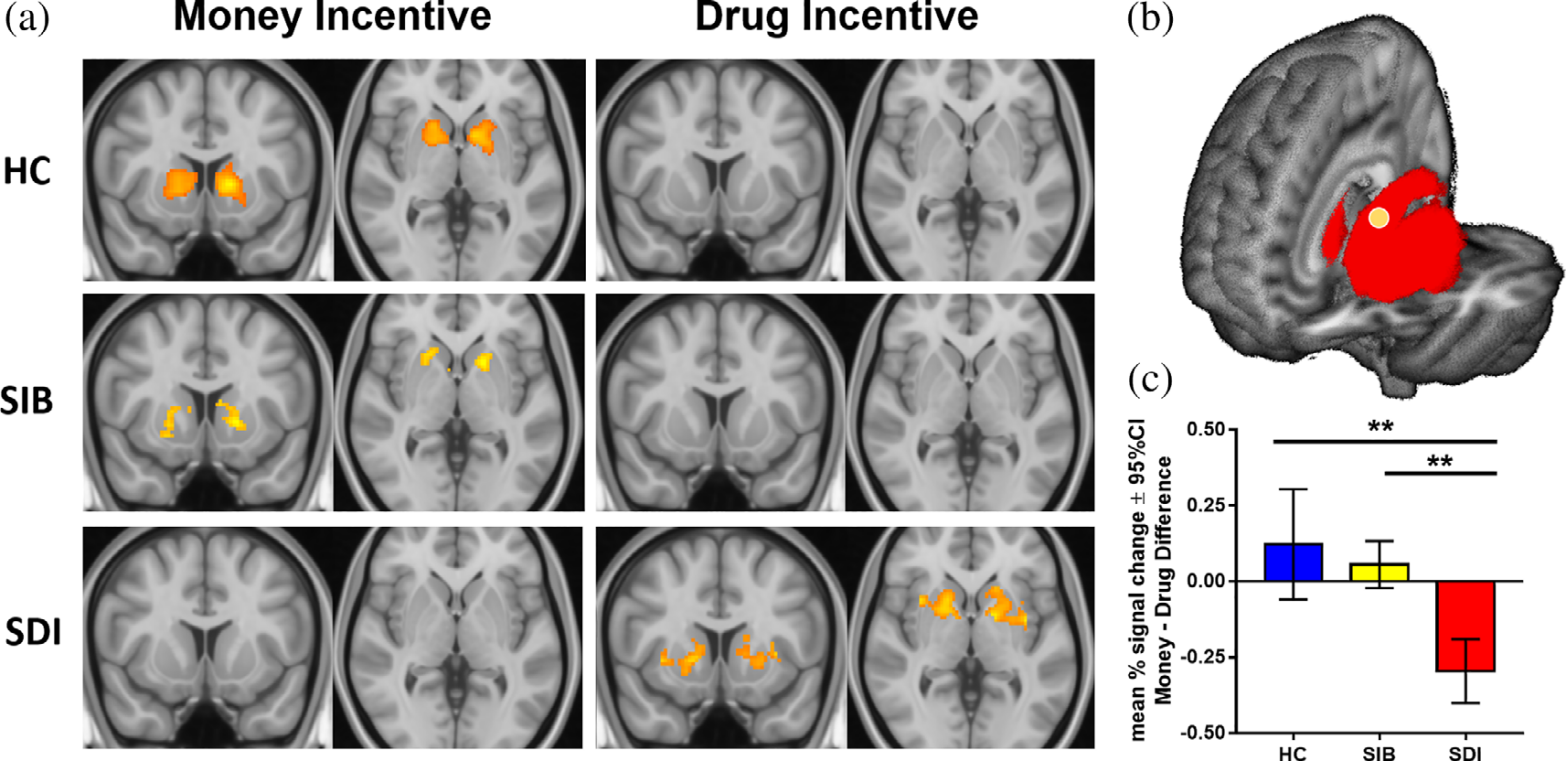
Striatum ROI activation during premature responding. (a) HC and SIB exhibit striatal activation during premature responding to money incentives, while SDI exhibit a similar pattern but with drug incentives (MNI [*y z*] = [10 0]). Activations in all groups span the caudate and putamen. (b) The striatum ROI (outlined in red), with the subregion in yellow in the anterior dorsal caudate (*n* = 6 voxels) exhibiting an opposing pattern with increased activation in both HC and SIB and decreased activation in SDI when responding prematurely to money versus drug incentives. (c) Mean percent signal change (with 95% CI) of the difference between activation for money versus drug incentives in the anterior dorsal caudate. A significant interaction, *F*(2, 123) = 5.99, *p* = .003, was found between group and incentive condition. Specifically, Money‐Drug difference was significantly greater in the HC than in SDI and in the SIB than in SDI (post‐hoc tests with false discovery rate correction, *p* = .0016 *p* = .0074, respectively). Asterisks in figure denote level of significance (**p < .01)

Interaction effects between groups (HC, SIB, and SDI) and the within‐subject factor of incentive block (money vs. drug) were tested in the striatum region of interest (ROI) as follows: for each participant, difference maps were calculated for contrast of parameter estimates between the premature > correct_money_ and premature > correct_drug_ contrasts. Using FSL randomize with *n* = 5,000 samples (Nichols & Holmes, 2003; Winkler, Ridgway, Webster, Smith, & Nichols, 2014), one sample t‐tests revealed the areas in which the groups showed greater activation to the premature > correct_money_ contrast than to the premature > correct_drug_ contrast. An F‐test comparing the three group means was used to detect any significant interaction between all three groups. This interaction was further investigated using independent sample t‐tests in FSL randomize (*n* = 5,000 samples), comparing the contrast of parameter estimates difference maps between the groups (HCs vs. SDIs, SIBs vs. SDIs) to indicate regions with a significant interaction (Figure 2). The resulting familywise‐error corrected p‐value maps with threshold‐free cluster enhancement (Smith & Nichols, 2009) from the striatum ROI were thresholded at *p* < .05 and the ROI comprising voxels with a significant interaction between both HCs and SDIs and SIBs and SDIs was selected for extraction of % signal change using featquery (http://mumford.fmripower.org/perchange_guide.pdf; https://fsl.fmrib.ox.ac.uk/fsl/fslwiki/FEAT/UserGuide#Featquery_-_FEAT_Results_Interrogation).

A potential limitation of the traditional mass univariate GLM in this instance is the low number of events (premature and corresponding correct responses, see Figure 1a) as explanatory variables of interest. To test whether the GLM findings were robust, we selected ROIs that were significantly active in group mean activation maps. We then extracted and preprocessed the timeseries of these ROIs, located the anticipation windows in which premature and correct responses were made and used the BOLD activation in each of these anticipation windows to predict whether this window included a premature or a correct response. If ROIs are more active during premature than during correct responses, their activation should be sufficient to classify response type. For this analysis, all correct responses were included. Inferior frontal cortex (IFC), anterior cingulate cortex (ACC), parietal operculum (pO), and striatal ROIs were chosen based on group mean activation. More details on timeseries extraction and preprocessing can be found in Supporting Information.

In HCs, the BOLD signal on each trial in the IFC, striatum, ACC, and parietal operculum (pO) was used to predict trial type (premature vs. correct) in money context, while the BOLD signal in the ACC was used to predict trial type in drug context. In the SIBs, the BOLD signal on each trial in the IFC, striatum, and ACC was used to predict trial type (premature vs. correct) in money context only. In the SDIs, BOLD signal on each trial in the IFC, ACC, and the pO was used to predict trial type in the money context, and BOLD signal in the IFC, ACC, striatum, and pO was used to predict trial type in drug context. These regions were significantly activated in the group mean maps in the GLM results and were thus selected to validate the group results. Hierarchical logistic regression with subject‐level random effects in RStudio (glmer function in the lme4 package) tested whether the ROI timeseries significantly predicted trial type. Receiver operating curves and area under the curve (roc and auc functions in the caTools package, plot and lines functions in the ggplot package) provided an additional metric of model performance.

#### 2.4.3 Brain‐behavior correlations

To assess possible brain‐behavior relationships, logistic regression models were fitted for each participant individually (using glm, MATLAB). Beta regression weight values for the IFC, striatum, and ACC were correlated with BIS‐11 (Patton, Stanford, & Barratt, 1995) and a self‐reported estimate of likelihood to pick up money on the floor (Value of Money). For SDIs, correlations were also assessed with the obsessive–compulsive drug use scale (OCDUS) scores. In the SDI group, ACC, striatum, and IFC beta values from the drug incentive condition were analyzed, whereas in the HC and SIB groups, ACC, striatum, and IFC values were used in the money incentive condition. Since the beta value distribution was non‐normal, Spearman's rank correlations with confidence intervals are reported, with p‐values using Bonferroni‐correction for multiple comparisons within each group.

#### 2.4.4 Dynamic causal modeling

To explore directional interactions (effective connectivity) between the regions identified by the mass univariate GLM analysis, a set of dynamic causal models were created, estimated, and tested in SPM12 (v6906). Following the GLM activations, dynamic causal models (DCM) were tested with the money incentive condition for the HC group and with the drug incentives for the SDI group. In two participants in each group the DCM analyses for three or more models failed to converge, resulting in these participants being excluded for this analysis. During the DCM analyses for the SIB group, the Bayesian model selection (BMS) phase did not yield a single winning model limiting any interpretation of their results. Additional information is reported in the supplementary information. The network of interest included three ROIs: IFC (pars opercularis), Caudate and ACC, in keeping with the recommendation that only commonly activated regions in both groups be included as nodes (Seghier et al., 2010). Time series were extracted in similar way to the logistic regression analysis, that is, based on the individual peak activations in response to the premature > correct contrast. Here, time series preprocessing only included despiking (>4*SD* from the mean) of the SDI group to address severe motion artifacts and were not mean centered (since the Eigenvariate extraction results in a mean of 0 and an *SD* of 1). First level GLM analyses were re‐estimated in SPM12 for the money and drug incentive conditions in a separate GLM and combined with the preprocessed timeseries in the DCM. Second level group maps estimated in SPM12 were consistent with 2nd level group maps estimated in FSL.

**Figure 3:**
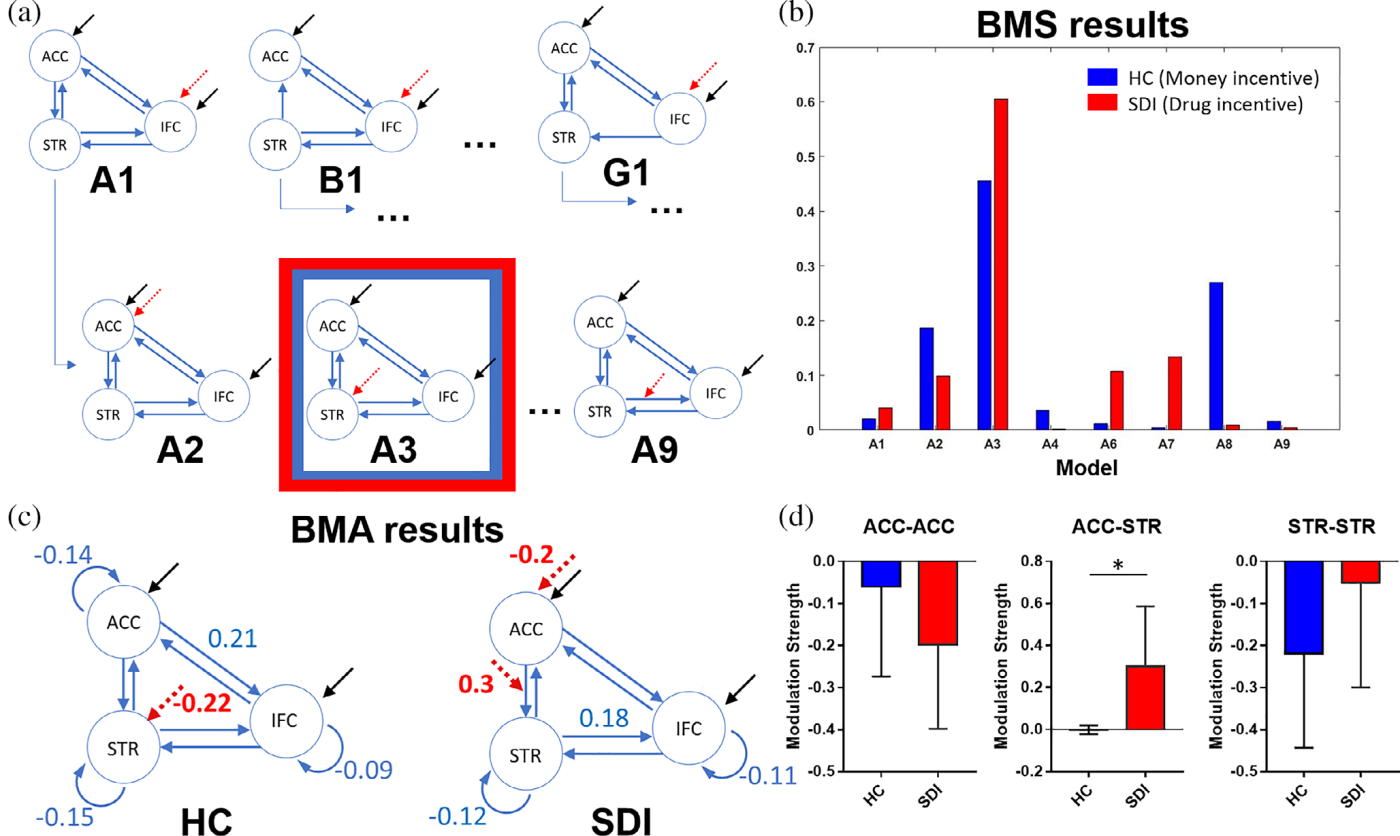
DCM connectivity analyses. (a) Model space and structure of the DCMs compared. Top row shows the 7 families, testing different possible connections between the 3 regions. Black arrows denote driving inputs (all MID trials) and red arrows indicate the modulatory effect of premature responding (premature vs. correct). Within the fully interactive family A, model A3 with modulation to the striatum is highlighted as having the greatest exceedance probabilities for both HC with money incentive and for SDI with drug incentives (see also Appendix S3). (b) Random effects Bayesian Model Selection within family A, demonstrating the evidence in support of model A3 for both groups, each with their relevant incentive. (c) Average connectivity estimates. All fixed connections survive Bonferroni corrections. Modulatory connections are significant at *p* < .05, uncorrected. (d) Mean group modulation strength estimates (with 95% CI) from the BMA for HC and SDI (each with their respective incentive). Only the ACC to striatum modulation differed significantly between the groups. Asterisk in figure denotes level of significance (*p < .05, uncorrected); ACC: anterior cingulate; STR: striatum incorporating caudate and putamen subregions that were active in the HC and SDI group, respectively; IFC: inferior frontal cortex including the frontal operculum only

Model space definition aimed to address two issues: firstly, we wanted to confirm the interactive architecture of the ACC—Caudate—IFC network by comparing a fully interactive model family (family A, Figure 3a) with architectures where one of the connections is pruned (model families B‐G). Secondly, we wanted to investigate at which node or connection in the network the modulation by premature>correct contrast occurs (models A1–A9). All principle experimental conditions (correct, premature, failure) were used as driving inputs to the ACC and IFC, similarly to previous DCM analyses of response inhibition (Rae et al., 2016; Rae, Hughes, Anderson, & Rowe, 2015). Modulatory effects were placed at each possible node and connection. Random effects (RFX) Bayesian model selection was ran on the full model space including families A–G, and on family A only; with both yielding the same results. Since strong a priori evidence from functional and structural connectivity studies (Choi, Tanimura, Vage, Yates, & Haber, 2017; d'Acremont, Fornari, & Bossaerts, 2013; Menon, 2015; Sadaghiani & D'Esposito, 2015; Uddin, 2016) and family comparison results supported the fully interactive model, the comparisons reported focus on models A1–A9. We computed exceedance probabilities (EPs) for each model and used those to determine the winning model (family) in a one‐state, bilinear, deterministic DCM. Bayesian model averaging (BMA) provided estimates of the fixed and modulatory connections in each subject, weighted by the evidence of each model tested (A1–A9). Using individual parameter estimates, group mean activations and group comparisons were tested using one sample *t*‐tests and independent sample t‐tests (Figure 3).

## 3 RESULTS

### 3.1 SDIs exhibit greater premature responding to drug incentives

Participants in all three groups occasionally made ‘premature’ responses in the anticipation phase while waiting for the target to appear unpredictably (Figure 1a). There were significant differences between HCs, SIBs, and SDIs in the number of premature responses for drug‐related incentives using the nonparametric Kruskal‐Wallis test (*χ* ^2^[2] = 10.56, *p* = .005, Figure 1b). The groups did not significantly differ for money incentives (*χ* ^2^[2] = 3.39, *p* = .18). Additionally, there were no significant group differences in performance accuracy for either money (*F*_2,129_ = 2.83, *p* = .06) or drug incentives (*F*_2,129_ = 0.63, *p* = .53). Mean (SD) numbers of correct trials for HCs, SIBs, and SDIs were 41(4), 41(4), and 39(5), respectively in the money context, and 41(5), 41(4), and 40(5), respectively in the drug context. The behavioral results indicate that while infrequent (9% of total responses), premature responding is ubiquitous, exhibited by the majority of participants. SDIs exhibited elevated levels, specifically with drug‐related incentives, with no differences between HCs and SIBs. This is consistent with greater self‐reported general impulsivity in SDI (Table 1), while capturing their behavioral difficulties in action‐restraint when anticipating drug‐related rewards.

### 3.2 Brain activation associated with premature responding depends on group‐specific incentives

We next investigated whole‐brain group activations associated with premature responding. To this end, trials where a premature response was detected were contrasted with adjacent trials with a correct response. fMRI analyses of the premature>correct contrast revealed wide‐spread incentive‐specific neural correlates of premature responses in the three groups. Group level activations (Figure 1c) for the money incentive condition in HCs and SIBs and for the drug incentive condition in SDIs included the inferior frontal cortex *pars opercularis* (IFC), dorsal anterior cingulate cortex (dACC), and dorsal striatum (dStriatum encompassing the caudate and putamen). For money incentives, the HC and SDI groups showed activation in the parietal operculum and the thalamus in addition to the dACC and IFC. For drug incentives, SDIs also activated portions of the motor, temporal, parietal cortices and the thalamus (Figure 1c, one sample t‐tests, cluster forming threshold *z* > 2.3, *p* < .05).

These findings suggest a similar pattern for the three groups with money incentives, but with drug incentives only SDIs exhibited activations associated with restraint failures. To assess this interpretation, group differences were interrogated using a whole brain mask. This revealed no significant differences in the monetary incentive condition. In contrast, in the drug condition SDIs showed greater activation than SIBs and HCs in the IFC, striatum, primary motor cortex, PCC, parietal, temporal areas and the thalamus in addition to the ventromedial PFC (SDI > HC only) and amygdala (SDI > HC only) (independent samples t‐tests, cluster forming threshold *z* > 2.3, voxelwise FWE corrected *p* < .05, Table S1). The BOLD results thus converge with the behavioral findings, with SDI showing clear abnormalities with drug incentives, and with SIBs differing from SDIs and being no different from controls.

If the BOLD activity were dependent on personal relevance regardless of incentive, then considerable overlap would be expected between money incentives in the HC and SIBs, and drug incentives in the SDI group. Conjunction analyses (fsl easythresh_conj) indeed confirmed large‐scale overlap in the IFC, dACC, striatum, and parietal operculum between the HCs and SIBs (money incentives) and SDIs (money and drug incentives). We can therefore conclude that across three separate groups, the findings specify a set of fronto‐striatal regions that are involved in impulsive responding when anticipating a personally‐desired outcome. That similar regions were activated in the drug users, regardless of incentive type, suggests a general system that is associated with failures in self‐restraint that is also sensitive to motivational processes.

### 3.3 BOLD activation in the IFC, dACC, dStriatum, and parietal operculum predicts trial type, thus validating the GLM results

To ascertain whether the GLM group mean activation maps in the three groups were robust to the limited number of premature trials, we assessed whether the resulting BOLD activations could reliably predict trial type. Specifically, BOLD timeseries activation in the IFC (*pars opercularis*), dACC, dStriatum, and the parietal operculum were used to predict whether trials were correct or premature (Figure 1d). Hierarchical logistic regression models with subject‐level random intercepts performed well in differentiating premature from correct responses in the money context (AUC_HC_ = 0.77; AUC_SIB_ = 0.73; AUC_SDI_ = 0.75) and in the drug context (AUC_SDI_ = 0.71). The differential contributions of each region of interest (ROI) to the prediction analyses can be found in Table S2. The results for financial incentives, found independently in the three groups, further lends support to the reliability of the involvement of these regions in premature responding. This, together with the considerable overlap between groups noted above supports and validates our fMRI findings.

### 3.4 Differential dStriatum involvement in SDI compared to HC and SIB

Previous work has established striatal involvement in addiction, task control and reward processing (Everitt & Robbins, 2016; Oldham et al., 2018). We thus focused on a ROI encompassing bilaterally the caudate, putamen, and nucleus accumbens. To assess the selective involvement of the striatum in failures of restraint specifically to drug cues in SDIs, interaction effects between group (SDI vs. SIB vs. HC) and incentive type (money vs drug) were examined within this striatal mask. Significant interaction effects in the caudate (MNI [−11 10 16], *t* = 3.2, *P*_FWE_ = 0.05), and putamen (MNI [−34 −2 −4], *t* = 3.9, *P*_FWE_ < 0.04) were driven by greater activation to money cues compared to drug cues in the HCs versus the SDIs (Figure S2). Similarly, interactive effects in the caudate (MNI [−12 12 16], *t* = 3.26, *P*_FWE_ = 0.04) and putamen (MNI [−34 −2 −4], *t* = 4.1, *P*_FWE_ = 0.01) were driven by greater activation to money cues compared to drug cues in the SIBs versus the SDIs. Figure 2b shows a subregion of the caudate, in which SDIs show a different pattern of activation from *both* SIBs and HCs (MNI [−12 12 16], HC vs SDI *t* = 3.31, *P*_FWE_ = 0.05; SIB vs SDI *t* = 3.26, *P*_FWE_ = 0.04; region extent 6 voxels). This more stringent and focused approach indicated that the BOLD response in the dStriatum was differentially sensitive to impulsive responding for money in non‐dependent individuals and to impulsive responding to drug cues in SDIs, regardless of familial vulnerability (Figure 2c). Overall, there was a clear opposing pattern in the dStriatum with reduced activation for monetary incentives in SDIs compared to the other two groups, pointing to specific abnormalities in this region in the SDIs.

### 3.5 Limited associations between traits, behavior and BOLD signal

We next investigated the relationship between neural activation associated with premature responses and self‐reported impulsivity (Barratt Impulsivity Scale; BIS‐11), money valuation rating (Value of Money), and in SDIs also compulsive drug use (OCDUS). For dACC, IFC and striatal regions, we used corresponding beta values from individual logistic regression models as a measure of each region's sensitivity to premature responses. In SDIs responding to drug incentives, caudate activation was positively associated with self‐reported impulsivity (*r*_s_ = 0.36, *p* = .02, 95%CI [0.06 0.6]) and with compulsive drug use (*r*_s_ = 0.40, *p* = .011, 95%CI [0.1 0.63]). In this group IFC beta values in the drug context were also significantly associated with impulsivity (*r*_s_ = 0.36, *p* = .02, 95%CI [0.06 0.6]), and with compulsive drug use (*r*_s_ = 0.42, *p* = .008, 95%CI [0.14 0.64]). In HCs, there was a significant negative association between caudate beta values for monetary incentives and money valuation ratings (*r*_s_ = −0.36, *p* = .02, 95%CI [−0.1–0.6]). No correlations survived Bonferroni correction, with nine comparisons in SDIs and six in the HCs, and hence are reported within an exploratory framework. Finally, no significant correlations were found in SIBs. While only suggestive, these correlations appear consistent with the interpretation above of an abnormal involvement of the striatum in impulsive responding in SDIs.

### 3.6 Group differences in effective connectivity in the same network

Although HCs and SDIs showed activation in similar brain regions when failing to restrain responses to different incentives, it is possible that the underlying network architecture and their connectivity differ in some way. This would be consistent with evidence positing deficient communication between prefrontal and subcortical regions in addiction (Goldstein & Volkow, 2011; Ma et al., 2015). Namely, the direction of effective connectivity between regions or the influence of response type (premature vs correct) on connectivity may differ between the two groups. To test these possibilities, we used DCMs based on neurobiologically plausible circuits (Choi, Tanimura, et al., 2017; Haber, 2016). Such DCMs fit generative models to assess the directed influence of one region over another, allowing us to compare competing hypotheses about functional interactions in a set of ROIs.

First, we assessed the underlying functional architecture of the network including the IFC, dACC, and striatum in the SDI (drug context) and HC (money context) groups. We tested models with either a pruned connection or the full model yielding seven model families. Within each family we varied all possible modulation locations, yielding 57 models in total (Figure 3a). These models were estimated and the evidence for each family was compared using family‐wise Bayesian Model Selection (BMS). For both groups independently, the fully interactive model architecture, including bidirectional connections between all regions of interest, was confirmed by family‐wise BMS (exceedance probabilities >.99). This is consistent with the literature reporting the presence of structural and functional connections between these regions (Choi, Tanimura, et al., 2017; Haber, 2016).

Additionally, we interrogated which connections were modulated by trial type. Focusing on the fully‐interacting model architecture of family A, BMS on models differing in the location of the modulation (IFC, dACC, dorsal striatum nodes, or one of the six directed connections) revealed the same winning model for the HC group network active in money context as for the SDI group network active in drug context (Figure 3b). Therefore, both HC and SDI participants appeared to activate the same network, provided that they find themselves in the appropriate incentive condition (money or drug, respectively). At the same time, exceedance probabilities of the winning models (Figure 3b), were below .9 (protected exceedance *p* = .14 and *p* = .21 for HCs and SDIs, respectively), suggesting some heterogeneity in the location of the modulation in both HC and SDI groups.

Next, the strength of the modulatory effects of the task (premature > correct trial type) and the fixed connections between ROIs were explored within each group using one‐sample *t*‐tests. We also questioned whether the coupling parameters of the network were different in the two groups, using independent sample *t*‐tests. Bayesian model averaging (BMA) allowed us to compute means for each model parameter, weighted by the posterior probability of each model for each subject. A summary of all parameters, including fixed connections between ROIs and task modulatory effects can be seen in Figure 3c. In HCs, BMA revealed a negative modulation of striatal activity by trial type (*t*_39_ = 2.0, *p* = .03), while in SDIs trial type showed negative modulation of dACC activity (*t*_38_ = 2.0, *p* = .03) and positive modulation of the dACC to dStriatum projection (*t*_38_ = 2.1, *p* = .02). A dStriatum negative (autoinhibitory) fixed connection was thus amplified on premature trials in the HC group, whereas a dACC autoinhibitory connection and an excitatory connection from the dACC to the dStriatum was amplified on premature trials in the SDI group.

Independent sample t‐tests, comparing the groups in modulation strength, showed only a significant difference in modulation of the dACC to dStriatum projection by trial type, which was higher in SDIs than in HCs (*t*_77_ = 2.13, *p* = .036, Figure 3d). Fixed connections between nodes did not differ between the two groups (all *p*s > .05) except for ACC to ACC autoinhibitory connection, which was higher in HC than SDI (*t*_77_ = 2.36, *p* = .021).

To summarize, Bayesian model selection revealed a fully interactive model architecture given relevant incentives, with bidirectional connections between the IFC, dACC and dStriatum. Among these interactive models, the dStriatum is critically involved as it is modulated by the premature vs correct trial condition. Bayesian model averaging suggested differences between HC and SDIs, with task‐based modulation changing striatal self‐connectivity directly in HC, while in SDIs task modulation changed dACC‐striatal connectivity.

## 4 DISCUSSION

Our results integrate research on inhibitory response control and reward‐related processing, offering insight into how these manifest jointly in the human brain and relate to impulsivity. The findings point to the intrinsic importance of context and the nature of the relevant incentives in modulating inhibitory control processes, particularly in relation to drug addiction. We show that failure of response control when faced with anticipating rewards is underpinned by fronto‐striatal and cingulo‐opercular network activations, wherein dorsal striatum regions play a key role. By focusing on failures of restraint in the MID task, the study demonstrates for the first time how impulsive actions can be triggered by drug incentive cues in individuals addicted to stimulant drugs.

### 4.1 Shared networks for premature responding to personally salient cues

This study identified key brain regions associated with failures of restraint in the presence of reward in SDI, their unaffected siblings and HC. Robust activation of the cingulo‐opercular and fronto‐striatal network regions comprising dACC, IFC (pars opercularis), inferior parietal cortex (parietal operculum), striatum and thalamus were present in healthy individuals and an attenuated version of this network minus thalamic and parietal activation in the unaffected siblings with monetary incentives. Activations were also seen in response to failed impulse control in SDI for money and drug incentives. That all three groups independently exhibited a similar pattern reinforces its robustness.

The regions identified here are known to be differentially activated to errors across a variety of cognitive tasks and populations (Neta et al., 2015; Norman et al., 2019). This is in keeping with the notion that premature responses are brought about by failure of control processes. Analyses of failures when trying to stop a prepotent response implicate similar regions encompassing the IFC, ACC, dorsal caudate, and inferior parietal cortex (Whelan et al., 2012). At the same time, premature responding is of special interest as it is instigated in the presence of an incentive‐driven impulse, pointing to general error processing mechanisms transcending different motivational contexts.

At the group level the neural correlates of action restraint were intimately linked to the personal relevance of the incentives. Thus, subjective value across all incentive types elicited activation in the regions described above (Bartra, McGuire, & Kable, 2013). Subjective value here refers to individuals' neural and behavioral response rather than self‐reported valence or arousal ratings, which have previously been shown to diverge in studies of alcohol dependence (Nees, Diener, Smolka, & Flor, 2012). Using multiple incentives revealed that healthy controls and the unaffected siblings exhibited activations when responding prematurely to monetary incentives but not to drug‐related cues. SDI demonstrated similar activations but most robustly to drug‐related cues, where they further showed increased activation in brain areas that encode motivation and emotional salience (e.g., amygdala, OFC). Greater activation to drug incentives in these regions was accompanied by elevated premature responding in the active users, suggesting that incentive salience was driving this behavior, consistent with studies in experimental animals and in other addiction‐related clinical populations (Dalley & Ersche, 2019; Voon, 2014).

### 4.2 Neural correlates of impulsivity in drug addiction

Increased impulsive behavior and corresponding increased activation in the presence of drug cues fit well within the iRISA framework (Goldstein & Volkow, 2002; Goldstein & Volkow, 2011; Zilverstand et al., 2018). Drug cues such as those used in our version of the MID task are known to have abnormally high motivational significance to cocaine users (Goldstein et al., 2008) and elicit increased approach behaviors and upregulation across the brain including the cingulo‐opercular network (Zilverstand et al., 2018). These networks are generally underactive in active stimulant users during standard cognitive task performance including inhibitory control processing (Morein‐Zamir & Robbins, 2015; Zilverstand et al., 2018). While this is generally true of stimulant addiction, previous studies of reward anticipation in nicotine addiction (Bühler et al., 2010) suggest that dependent smokers can show similar levels of reactivity to cigarette compared to monetary rewards. Presently, whole brain analyses did not find significant hypoactivation in the SDI compared to control participants in whole‐brain analyses with monetary incentives. This is in keeping with the limited differences in task performance and function between these same individuals in monetary reward processing (Just et al., 2019) and consistent with the broader literature on processing of non‐drug rewards in addicted individuals (Zilverstand et al., 2018). While consistent with the notion that brain activity during inhibitory control is stimulus‐dependent (Czapla et al., 2017), this limits proposals that drug use diminishes the perceived value of non‐drug rewards to only specific brain regions (Goldstein & Volkow, 2011). Taken together the results support an imbalance between incentive salience of drug and non‐drug rewards in drug users (Bühler et al., 2010). The iRISA model will likely benefit from further refinement by taking into account the nature of the reactivity measures, including behavioral, neural, physiological, or subjective responses (Nees et al., 2012).

Nevertheless, the importance of incentive type to impulsive responding in addiction is reflected across the brain and most clearly by the activation of caudate and putamen subregions of the dorsal striatum. Here, not only was there greater activation in SDI in the presence of drug incentives, but there was indeed a blunted response in the presence of monetary incentives compared to either of the two other groups. The temporal and correlational features of fMRI preclude us from determining the exact nature of dorsal striatal involvement in the processing of premature responding. This region could be directly implicated in triggering the impulsive responding or could be linked to monitoring processes. The dorsal striatum is involved in action control (Graybiel, 1995; Haber, 2016), but is also activated when participants anticipate rewards (Oldham et al., 2018), with neurons integrating reward information with movement processing (Schultz, 2016). We therefore tentatively attribute the striatal involvement to the failure of control in the face of personally meaningful incentives. This explanation dovetails with the role of the dorsal striatum in canceling planned motor responses (Bari & Robbins, 2013; Eagle & Baunez, 2010) and impulsive choice (Kim & Im, 2018), while providing evidence for its involvement in additional aspects of impulsivity in humans.

Abnormal striatal processing in addiction has been consistently linked to general aberrations in cortico‐striatal circuitry that subserves motor, cognitive, and motivational processes (Choi, Ding, & Haber, 2017; Choi, Tanimura, et al., 2017). When considering this circuit, we show that SDIs and HCs share a strikingly similar network architecture, provided that a relative incentive is present. Nevertheless, while the striatum was consistently modulated by premature responding in the two groups, effective connectivity between the IFC, dACC, and striatum pointed to the differential involvement of the latter two regions. The dACC sits at the connectional intersection of the brain's reward and action networks (Haber, 2016), and is ideally situated to regulate impulsive behaviors. This region thus provides top‐down inhibitory control over striatal representations of action and stimulus values and has been found to over‐activate in SDIs with failures of response inhibition (Morein‐Zamir, Simon Jones, Bullmore, Robbins, & Ersche, 2013). Here the observed enhanced directed connectivity from dACC to dorsal striatum in SDIs relative to HCs suggests that interactions between the salience network and dorsal striatum via the ACC play a critical role in impulsive responding in drug abuse. Investigations using dynamic causal models in a response inhibition task also pointed to aberrant modulation of the dACC to dorsal striatum projections in cocaine dependent individuals (Ma et al., 2015). Present results converge to support deficient communication between prefrontal and subcortical regions in addiction (Goldstein & Volkow, 2011).

### 4.3 Additional implications of the present findings

Interactions between the PFC and striatum are also found in non‐human animal studies investigating PFC involvement in failures of restraint. The rodent prelimbic cortex, a homolog of human dACC in addition to rodent infralimbic cortex, a homolog of the human ventromedial PFC, has been implicated in premature responding (Dalley et al., 2011; Dalley & Robbins, 2017) and behavioral control (Gourley & Taylor, 2016). We observed ventromedial PFC activations in SDIs consistent with previous human and animal literature (Dalley et al., 2011; Morris et al., 2016), again reinforcing the importance of incentive salience in this group. We note that the ventral striatum did not appear to be uniquely activated by failures of restraint, though it was robustly associated in all groups with general reward anticipation (Just et al., 2019). Given its role in addiction, reward seeking, impulsivity and action initiation, striatal dopaminergic dysregulation may be a putative mechanism underlying the present findings in SDIs. Buckholtz and colleagues (2010) showed that impulsivity was associated with increased dopamine release in the striatum as a result of reduced D2R binding in the midbrain. Others have shown that increased levels of impulsivity are associated with low dopamine D2 receptors in the striatum in both healthy and drug addicted individuals (Lee et al., 2009; Trifilieff and Martinez, 2014). In chronic cocaine users elevated dopamine neurotransmission in the dorsal striatum specifically was noted in the presence of cocaine‐related cues (Volkow et al., 2006), dovetailing with its present involvement in incentive salience. Changes to dorsal striatum physiology involving D_2_ receptors have also been implicated in chronic exposure to cocaine in experimental animals (Porrino, Daunais, Smith, & Nader, 2004), consistent with its relationship to impaired impulsive control in the present study.

Assessing the unaffected siblings of SDI allowed us to test whether premature responding is a familial predisposition to stimulant drug addiction. Performance and neural correlates in siblings indicated this was not the case for both incentive types. As the connectivity analyses for the siblings did not result in a single winning model (see Figure S3) we could not extend this conclusion to network connectivity. Nevertheless, on balance we conclude that an impulsive endophenotype does not appear to extend to premature responding, at least under reward‐based conditions. Indeed, it may be that increased restraint served as a protective factor for the siblings. Alternatively, it remains possible that the ability to restrain responding may be compromised in unaffected family members under more constrained conditions, such as those eliciting negative urgency (Um, Whitt, Revilla, Hunton, & Cyders, 2019), with greater cognitive demands, or drug exposure (Sanchez‐Roige, Stephens, & Duka, 2016).

The MID task has been favored in reward research partly because it requires participants to make simple decisions, minimizing cognitive confounds (Oldham et al., 2018). This allowed us to attribute premature responses to failures of inhibitory control specifically. This approach offers a parsimonious measure of impulsive responding to diverse incentives in clinical and non‐clinical populations. The presence of a relatively wide anticipation window likely increased premature responding frequency overall and allowed greater individual variability. Future research may further explore how varying specific task parameters in the MID task such as the nature and timing of reward delivery, can elicit or minimize premature responding, testing convergence with preclinical models. Additionally, performance and network changes in at‐risk groups or following prolonged abstinence should be explored. This could better elucidate the exact role of various risk factors in addiction and point to potential mitigating factors.

In conclusion, our findings provide insight into the brain networks recruited during failures of restraint in a well‐characterized sample of SDIs, their unaffected siblings and HC. Limited cognitive control over action was evident in the SDI group, particularly involving incentive‐based situations in line with prominent theorizing. Whole brain analyses provide evidence for cortico‐striatal network disruption in addiction involving top‐down control by the PFC and its interactions with striatal structures with altered connectivity and abnormal striatal activation patterns. By capitalizing on the presence of premature responses in this version of the MID task and introducing a novel analysis, our results indicate that different forms of impulsive behaviors are comprised of separable, though often overlapping neural networks and are selectively modulated by incentive motivational processes.

## CONFLICT OF INTEREST

CM is supported by the Wellcome Trust (105602/Z/14/Z) and the NIHR Cambridge Biomedical Research Centre. PZ was supported by the Pinsent Darwin studentship from the Department of Physiology, Development and Neuroscience, University of Cambridge. The remaining authors declare no conflict of interests.

## Supporting information


**Appendix S1**: Supporting information

## Data Availability

Custom code used for the analyses in this article is publicly available at https://github.com/peterzhukovsky/fMRI_GLM_validation. The authors can also provide outputs of the MRI analyses upon reasonable request.

## References

[hbm24941-bib-0320] Balodis, I. M. , & Potenza, M. N. (2015). Anticipatory reward processingin addicted populations: A focus on the monetary incentive delay task. Biol Psychiatry, 77, 434–444. 10.1016/j.biopsych.2014.08.020 25481621 PMC4315733

[hbm24941-bib-0001] Bari, A. , & Robbins, T. W. (2013). Inhibition and impulsivity: Behavioral and neural basis of response control. Progress in Neurobiology, 108, 44–79. 10.1016/j.pneurobio.2013.06.005 23856628

[hbm24941-bib-0002] Bartra, O. , McGuire, J. T. , & Kable, J. W. (2013). The valuation system: A coordinate‐based meta‐analysis of BOLD fMRI experiments examining neural correlates of subjective value. NeuroImage, 25, 412–427. 10.1016/j.neuroimage.2013.02.063 PMC3756836 23507394

[hbm24941-bib-0319] Belin, D. , Mar, A. C. , Dalley, J. W. , Robbins, T. W. , & Everitt, B. J. (2008). High impulsivity predicts the switch to compulsive cocaine‐taking. Science, 320, 1352–1355. 10.1126/science.1158136 18535246 PMC2478705

[hbm24941-bib-0318] Berridge, K. C. (2007). The debate over dopamine's role inreward: The case for incentive salience. Psychopharmacology (Berl), 191, 391–431. 17072591 10.1007/s00213-006-0578-x

[hbm24941-bib-0003] Botvinick, M. , & Braver, T. (2015). Motivation and cognitive control: From behavior to neural mechanism. Annual Review of Psychology, 66, 83–113. 10.1146/annurev-psych-010814-015044 25251491

[hbm24941-bib-0004] Buckholtz, J. W. , Treadway, M. T. , Cowan, R. L. , Woodward, N. D. , Li, R. , Ansari, M. S. , … Zald, D. H. (2010). Dopaminergic network differences in human impulsivity. Science, 329, 532. 20671181 10.1126/science.1185778 PMC3161413

[hbm24941-bib-0005] Bühler, M. , Vollstädt‐Klein, S. , Kobiella, A. , Budde, H. , Reed, L. J. , Braus, D. F. , … Smolka, M. N. (2010). Nicotine dependence is characterized by disordered reward processing in a network driving motivation. Biological Psychiatry, 67, 745–752. 10.1016/j.biopsych.2009.10.029 20044075

[hbm24941-bib-0006] Choi, E. Y. , Ding, S.‐L. , & Haber, S. N. (2017). Combinatorial inputs to the ventral striatum from the temporal cortex, frontal cortex, and amygdala: Implications for segmenting the striatum. Eneuro, 4, ENEURO.0392‐17.2017. 10.1523/ENEURO.0392-17.2017 PMC5740454 29279863

[hbm24941-bib-0007] Choi, E. Y. , Tanimura, Y. , Vage, P. R. , Yates, E. H. , & Haber, S. N. (2017). Convergence of prefrontal and parietal anatomical projections in a connectional hub in the striatum. NeuroImage, 146, 821–832. 10.1016/j.neuroimage.2016.09.037 27646127 PMC5841917

[hbm24941-bib-0317] Cope, L. M. , Martz, M. E. , Hardee, J. E. , Zucker, R. A. , & Heitzeg, M. M. (2019). Reward activation in childhood predicts adolescent substance use initiation ina high‐risk sample. Drug Alcohol Depend, 194, 318–325. http://www.ncbi.nlm.nih.gov/pubmed/30471583 30471583 10.1016/j.drugalcdep.2018.11.003 PMC6540995

[hbm24941-bib-0008] Czapla, M. , Baeuchl, C. , Simon, J. J. , Richter, B. , Kluge, M. , Friederich, H. C. , … Loeber, S. (2017). Do alcohol‐dependent patients show different neural activation during response inhibition than healthy controls in an alcohol‐related fMRI go/no‐go‐task? Psychopharmacology, 234, 1001–1015. 28161772 10.1007/s00213-017-4541-9

[hbm24941-bib-0009] d'Acremont, M. , Fornari, E. , & Bossaerts, P. (2013). Activity in inferior parietal and medial prefrontal cortex signals the accumulation of evidence in a probability learning task. PLoS Computational Biology, 9, 1–11. 10.1371/journal.pcbi.1002895 PMC3561043 23401673

[hbm24941-bib-0316] Dalley, J. W. , Mar, A. C. , Economidou, D. , & Robbins, T. W. (2008). Neurobehavioral mechanisms of impulsivity: Fronto‐striatal systems andfunctional neurochemistry. Pharmacol Biochem Behav, 90, 250–260. 18272211 10.1016/j.pbb.2007.12.021

[hbm24941-bib-0010] Dalley, J. W. , & Ersche, K. D. (2019). Neural circuitry and mechanisms of waiting impulsivity: Relevance to addiction. Philosophical Transactions of the Royal Society of London. Series B, Biological Sciences, 374, 20180145. 30966923 10.1098/rstb.2018.0145 PMC6335458

[hbm24941-bib-0011] Dalley, J. W. , Everitt, B. J. , & Robbins, T. W. (2011). Impulsivity, compulsivity, and top‐down cognitive control. Neuron, 69, 680–694. 10.1016/j.neuron.2011.01.020 21338879

[hbm24941-bib-0012] Dalley, J. W. , & Robbins, T. W. (2017). Fractionating impulsivity: Neuropsychiatric implications. Nature Reviews. Neuroscience, 18, 158–171. 10.1038/nrn.2017.8 28209979

[hbm24941-bib-0013] Desikan, R. S. , Ségonne, F. , Fischl, B. , Quinn, B. T. , Dickerson, B. C. , Blacker, D. , … Killiany, R. J. (2006). An automated labeling system for subdividing the human cerebral cortex on MRI scans into gyral based regions of interest. NeuroImage, 31, 968–980. 16530430 10.1016/j.neuroimage.2006.01.021

[hbm24941-bib-0014] Eagle, D. M. , & Baunez, C. (2010). Is there an inhibitory‐response‐control system in the rat? Evidence from anatomical and pharmacological studies of behavioral inhibition. Neuroscience and Biobehavioral Reviews, 34, 50–72. 19615404 10.1016/j.neubiorev.2009.07.003 PMC2789250

[hbm24941-bib-0315] Ersche, K. D. , Barnes, A. , Jones, P. S. , Morein‐Zamir, S. , Robbins, T. W. , & Bullmore, E. T. (2011). Abnormal structure of frontostriatal brain systems is associated with aspects of impulsivity and compulsivity in cocaine dependence. Brain, 134, 2013–2024. 21690575 10.1093/brain/awr138 PMC3122375

[hbm24941-bib-0314] Ersche, K. D. , Jones, P. S. , Williams, G. B. , Turton, A. J. , Robbins, T. W. , & Bullmore, E. T. (2012a). Abnormal brain structure implicated in stimulant drug addiction. Science, 335, 601–604. 22301321 10.1126/science.1214463

[hbm24941-bib-0015] Ersche, K. D. , Turton, A. J. , Chamberlain, S. R. , Müller, U. , Bullmore, E. T. , & Robbins, T. W. (2012b). Cognitive dysfunction and anxious‐impulsive personality traits are endophenotypes for drug dependence. The American Journal of Psychiatry, 169, 926–936. 22952072 10.1176/appi.ajp.2012.11091421 PMC3533378

[hbm24941-bib-0313] Ersche, K. D. , Williams, G. B. , Robbins, T. W. , & Bullmore, E. T. (2013). Meta‐analysis of structural brain abnormalities associated with stimulant drug dependence and neuroimaging of addiction vulnerability and resilience. Curr Opin Neurobiol, 23, 615–624. 10.1016/j.conb.2013.02.017 23523373

[hbm24941-bib-0016] Evenden, J. L. (1999). Varieties of impulsivity. Psychopharmacology, 146, 348–361. 10550486 10.1007/pl00005481

[hbm24941-bib-0017] Everitt, B. J. , & Robbins, T. W. (2016). Drug addiction: Updating actions to habits to compulsions ten years on. Annual Review of Psychology, 67, 23–50. 10.1146/annurev-psych-122414-033457 26253543

[hbm24941-bib-0018] Goldstein, R. Z. , Woicik, P. A. , Moeller, S. J. , Telang, F. , Jayne, M. , Wong, C. , … Volkow, N. D. (2008). Liking and wanting of drug and non‐drug rewards in active cocaine users: The STRAP‐R questionnaire. Journal of Psychopharmacology, 24, 257–266. 18801822 10.1177/0269881108096982 PMC2820142

[hbm24941-bib-0019] Goldstein, R. Z. , & Volkow, N. D. (2011). Dysfunction of the prefrontal cortex in addiction: Neuroimaging findings and clinical implications. Nature Reviews. Neuroscience, 12, 652–669. 10.1038/nrn3119 22011681 PMC3462342

[hbm24941-bib-0020] Goldstein, R. Z. , & Volkow, N. D. (2002). Drug addiction and its underlying neurobiological basis: Neuroimaging evidence for the involvement of the frontal cortex. The American Journal of Psychiatry, 159, 1642–1652. 12359667 10.1176/appi.ajp.159.10.1642 PMC1201373

[hbm24941-bib-0021] Gourley, S. L. , & Taylor, J. R. (2016). Going and stopping: Dichotomies in behavioral control by the prefrontal cortex. Nature Neuroscience, 19, 656–664. 29162973 10.1038/nn.4275 PMC5087107

[hbm24941-bib-0022] Graybiel, A. M. (1995). Building action repertoires: Memory and learning functions of the basal ganglia. Current Opinion in Neurobiology, 5, 733–741. 8805417 10.1016/0959-4388(95)80100-6

[hbm24941-bib-0023] Haber, S. N. (2016). Corticostriatal circuitry. Neurosci 21st Century From Basic to Clin Second Ed:1721–1741.

[hbm24941-bib-0024] Hull, C. L. (1943). Principles of Behavior: An Introduction to Behavior Theory. D. Appleton‐Century Company, Inc. Vol. 4118.

[hbm24941-bib-0025] James, W. (1890). Principles of Psychology.

[hbm24941-bib-0026] Jenkinson, M. , Bannister, P. , Brady, M. , & Smith, S. (2002). Improved optimization for the robust and accurate linear registration and motion correction of brain images. NeuroImage, 17, 825–841. 12377157 10.1016/s1053-8119(02)91132-8

[hbm24941-bib-0027] Just, A. L. , Meng, C. , Smith, D. G. , Bullmore, E. T. , Robbins, T. W. , & Ersche, K. D. (2019). Effects of familial risk and stimulant drug use on the anticipation of monetary reward: An fMRI study. Translational Psychiatry, 9, 65 10.1038/s41398-019-0399-4 30718492 PMC6362203

[hbm24941-bib-0312] Koob, G. F. , & Le Moal, M. (2005). Plasticity of reward neurocircuitry and the ‘dark side’ of drug addiction. Nature, 8, 1442–1444. 10.1038/nn1105-1442 16251985

[hbm24941-bib-0028] Kim, B. S. , & Im, H. I. (2018). The role of the dorsal striatum in choice impulsivity. Annals of the New York Academy of Sciences, 1451, 92–111. 30277562 10.1111/nyas.13961

[hbm24941-bib-0029] Kjome, K. L. , Lane, S. D. , Schmitz, J. M. , Green, C. , Ma, L. , Prasla, I. , … Moeller, F. G. (2010). Relationship between impulsivity and decision making in cocaine dependence. Psychiatry Research, 178, 299–304. 10.1016/j.psychres.2009.11.024 20478631 PMC2904056

[hbm24941-bib-0311] Lee, B. , London, E. D. , Poldrack, R. A. , Farahi, J. , Nacca, A. , … Mandelkern, M. A. (2009). Striatal dopamine D2/D3 receptor availability is reduced in methamphetamine dependence and is linked to impulsivity. Journal of Neuroscience, 29, 14734–14740. 19940168 10.1523/JNEUROSCI.3765-09.2009 PMC2822639

[hbm24941-bib-0310] Luijten, M. , Schellekens, A. F. , Kühn, S. , Machielse, M. W. J. , & Sescousse, G. (2017). Disruption of reward processing in addiction: an image‐based meta‐analysis of functional magnetic resonance imaging studies. JAMA Psychiatry, 74, 387–398. 28146248 10.1001/jamapsychiatry.2016.3084

[hbm24941-bib-0309] Ma, L. , Steinberg, J. L. , Hasan, K. M. , Narayana, P. A. , Kramer, L. A. , & Moeller, F. G. (2014). Stochastic dynamic causal modeling of working memory connections in cocaine dependence. Human Brain Mapping, 35, 760–778. 23151990 10.1002/hbm.22212 PMC4440319

[hbm24941-bib-0030] Ma, L. , Steinberg, J. L. , Cunningham, K. A. , Lane, S. D. , Bjork, J. M. , Neelakantan, H. , … Moeller, F. G. (2015). Inhibitory behavioral control: A stochastic dynamic causal modeling study comparing cocaine dependent subjects and controls. NeuroImage Clinical, 7, 837–847. 26082893 10.1016/j.nicl.2015.03.015 PMC4459041

[hbm24941-bib-0308] Mechelmans, D. J. , Strelchuk, D. , Doñamayor, N. , Banca, P. , Robbins, T. W. , Baek, K. , & Voon, V. (2017). Reward sensitivity and waiting impulsivity: Shift towards reward valuation away from action control. Int J Neuropsychopharmacol, 20, 971–978. 29020291 10.1093/ijnp/pyx072 PMC5716204

[hbm24941-bib-0031] Menon, V. (2015). Salience Network In: Arthur W. Toga , editor. Brain Mapping: An Encyclopedic Reference, vol. 2, pp. 597‐611. Philadelphia, USA: Academic Press, Elsevier 10.1016/B978-0-12-397025-1.00052-X

[hbm24941-bib-0032] Morein‐Zamir, S. , & Robbins, T. W. (2015). Fronto‐striatal circuits in response‐inhibition: Relevance to addiction. Brain Research, 1628, 117–129. 10.1016/j.brainres.2014.09.012 25218611 PMC4686018

[hbm24941-bib-0033] Morein‐Zamir, S. , Simon Jones, P. , Bullmore, E. T. , Robbins, T. W. , & Ersche, K. D. (2013). Prefrontal hypoactivity associated with impaired inhibition in stimulant‐dependent individuals but evidence for hyperactivation in their unaffected siblings. Neuropsychopharmacology, 38, 1945–1953. 10.1038/npp.2013.90 23609131 PMC3746700

[hbm24941-bib-0034] Morris, L. S. , Kundu, P. , Baek, K. , Irvine, M. A. , Mechelmans, D. J. , Wood, J. , … Voon, V. (2016). Jumping the gun: Mapping neural correlates of waiting impulsivity and relevance across alcohol misuse. Biological Psychiatry, 79, 499–507. 10.1016/j.biopsych.2015.06.009 26185010 PMC4764648

[hbm24941-bib-0035] Nees, F. , Diener, C. , Smolka, M. N. , & Flor, H. (2012). The role of context in the processing of alcohol‐relevant cues. Addiction Biology, 17, 441–451. 21790904 10.1111/j.1369-1600.2011.00347.x

[hbm24941-bib-0036] Neta, M. , Miezin, F. M. , Nelson, S. M. , Dubis, J. W. , Dosenbach, N. U. F. , Schlaggar, B. L. , & Petersen, S. E. (2015). Spatial and temporal characteristics of error‐related activity in the human brain. The Journal of Neuroscience, 35, 253–266. 10.1523/JNEUROSCI.1313-14.2015 25568119 PMC4287146

[hbm24941-bib-0037] Nichols, T. , & Holmes, A. (2003). Nonparametric permutation tests for functional neuroimaging. Human Brain Function Second Edition, 25, 887–910. 10.1002/hbm.1058 PMC6871862 11747097

[hbm24941-bib-0038] Norman, L. J. , Taylor, S. F. , Liu, Y. , Radua, J. , Chye, Y. , De Wit, S. J. , … Fitzgerald, K. (2019). Error processing and inhibitory control in obsessive‐compulsive disorder: A meta‐analysis using statistical parametric maps. Biological Psychiatry, 85, 713–725. 10.1016/j.biopsych.2018.11.010 30595231 PMC6474799

[hbm24941-bib-0039] Oldham, S. , Murawski, C. , Fornito, A. , Youssef, G. , Yücel, M. , & Lorenzetti, V. (2018). The anticipation and outcome phases of reward and loss processing: A neuroimaging meta‐analysis of the monetary incentive delay task. Human Brain Mapping, 39, 3398–3418. 29696725 10.1002/hbm.24184 PMC6055646

[hbm24941-bib-0040] Patton, J. H. , Stanford, M. S. , & Barratt, E. S. (1995). Factor structure of the barratt impulsiveness scale. Journal of Clinical Psychology, 51, 768–774. 8778124 10.1002/1097-4679(199511)51:6<768::aid-jclp2270510607>3.0.co;2-1

[hbm24941-bib-0306] Peña‐Oliver, Y. , Carvalho, F. M. , Sanchez‐Roige, S. , Quinlan, E. B. , Jia, T. , … IMAGEN Consortium . (2016). Mouse and human genetic analyses associate kalirin with ventral striatal activation during impulsivity and with alcohol misuse. Front Genet, 7, 52. 27092175 10.3389/fgene.2016.00052 PMC4823271

[hbm24941-bib-0041] Porrino, L. J. , Daunais, J. B. , Smith, H. R. , & Nader, M. A. (2004). The expanding effects of cocaine: Studies in a nonhuman primate model of cocaine self‐administration. Neuroscience and Biobehavioral Reviews, 27, 813–820. 15019430 10.1016/j.neubiorev.2003.11.013

[hbm24941-bib-0042] Rae, C. L. , Nombela, C. , Rodríguez, P. V. , Ye, Z. , Hughes, L. E. , Jones, P. S. , … Rowe, J. B. (2016). Atomoxetine restores the response inhibition network in Parkinson's disease. Brain, 139, 2235–2248. 27343257 10.1093/brain/aww138 PMC4958901

[hbm24941-bib-0043] Rae, C. L. , Hughes, L. E. , Anderson, M. C. , & Rowe, J. B. (2015). The prefrontal cortex achieves inhibitory control by facilitating subcortical motor pathway connectivity. The Journal of Neuroscience, 35, 786–794. 25589771 10.1523/JNEUROSCI.3093-13.2015 PMC4293423

[hbm24941-bib-0305] Robbins, T. W. , Gillan, C. M. , Smith, D. G. , de Wit, S. , & Ersche, K. D. (2012). Neurocognitive endophenotypes of impulsivity and compulsivity: Towards dimensional psychiatry. Trends Cogn Sci, 16, 81–91. 22155014 10.1016/j.tics.2011.11.009

[hbm24941-bib-0044] Sadaghiani, S. , & D'Esposito, M. (2015). Functional characterization of the cingulo‐opercular network in the maintenance of tonic alertness. Cerebral Cortex, 25, 2763–2773. 24770711 10.1093/cercor/bhu072 PMC4537431

[hbm24941-bib-0304] Sanchez‐Roige, S. , Baro, V. , Trick, L. , Peñ A‐Oliver, Y. , Stephens, D. N. , & Duka, T. (2014). Exaggerated waiting impulsivity associated with human binge drinking, and high alcohol consumption in mice. Neuropsychopharmacology, 39, 2919–2927. 24947901 10.1038/npp.2014.151 PMC4229569

[hbm24941-bib-0045] Sanchez‐Roige, S. , Stephens, D. N. , & Duka, T. (2016). Heightened impulsivity: Associated with family history of alcohol misuse, and a consequence of alcohol intake. Alcoholism, Clinical and Experimental Research, 40, 2208–2217. 10.1111/acer.13184 27565012

[hbm24941-bib-0046] Schultz, W. (2016). Reward functions of the basal ganglia. Journal of Neural Transmission, 123, 679–693. 26838982 10.1007/s00702-016-1510-0 PMC5495848

[hbm24941-bib-0303] Seghier, M. L. , Zeidman, P. , Neufeld, N. H. , Leff, A. P. , & Price, C. J. (2010). Identifying abnormal connectivity in patients using dynamic causal modeling off MRI responses. Front Syst Neurosci, 4, 1–14. 20838471 10.3389/fnsys.2010.00142 PMC2936900

[hbm24941-bib-0047] Smith, S. M. , & Nichols, T. E. (2009). Threshold‐free cluster enhancement: Addressing problems of smoothing, threshold dependence and localisation in cluster inference. NeuroImage, 44, 83–98. 10.1016/j.neuroimage.2008.03.061 18501637

[hbm24941-bib-0048] Smith, S. M. (2002). Fast robust automated brain extraction. Human Brain Mapping, 17, 143–155. 12391568 10.1002/hbm.10062 PMC6871816

[hbm24941-bib-0302] Swick, D. , Ashley, V. , & Turken, U. (2011). Are the neural correlates of stopping and not going identical? Quantitative meta‐analysis of two response inhibition tasks. Neuroimage, 56, 1655–1665. 10.1016/j.neuroimage.2011.02.070 21376819

[hbm24941-bib-0049] Trifilieff, P. , & Martinez, D. (2014). Imaging addiction: D 2 receptors and dopamine signaling in the striatum as biomarkers for impulsivity. Neuropharmacology, 76, 498–509. 23851257 10.1016/j.neuropharm.2013.06.031 PMC4106030

[hbm24941-bib-0050] Uddin, L. Q. (2016). Anatomy of the salience network. Salience Network of the Human Brain. 10.1016/B978-0-12-804593-0.00002-3

[hbm24941-bib-0051] Um, M. , Whitt, Z. T. , Revilla, R. , Hunton, T. , & Cyders, M. A. (2019). Shared neural correlates underlying addictive disorders and negative urgency. Brain Sciences, 9, 1–17. 10.3390/brainsci9020036 PMC6406305 30744033

[hbm24941-bib-0052] Vigil‐Colet, A. (2007). Impulsivity and decision making in the balloon analogue risk‐taking task. Personality and Individual Differences, 43, 37–45.

[hbm24941-bib-0053] Volkow, N. D. , Wang, G.‐J. , Telang, F. , Fowler, J. S. , Logan, J. , Childress, A.‐R. , … Wong, C. (2006). Cocaine cues and dopamine in dorsal striatum: Mechanism of craving in cocaine addiction. The Journal of Neuroscience, 26, 6583–6588. 16775146 10.1523/JNEUROSCI.1544-06.2006 PMC6674019

[hbm24941-bib-0054] Voon, V. (2014). Models of impulsivity with a focus on waiting impulsivity: Translational potential for neuropsychiatric disorders. Current Addiction Reports, 1, 281–288. 25346881 10.1007/s40429-014-0036-5 PMC4201744

[hbm24941-bib-0301] Voon, V. , Chang‐Webb, Y. C. , Morris, L. S. , Cooper, E. , Sethi, A. , Baek, K. , … Harrison, N. A. (2016). Waiting impulsivity: The influence of acute methylphenidate and feedback. Int J Neuropsychopharmacol, 19, 1–10. 10.1093/ijnp/pyv074 PMC4772268 26136351

[hbm24941-bib-0055] Whelan, R. , Conrod, P. J. , Poline, J.‐B. , Lourdusamy, A. , Banaschewski, T. , Barker, G. J. , … Consortium the I . (2012). Adolescent impulsivity phenotypes characterized by distinct brain networks. Nature Neuroscience, 15, 920–925. 22544311 10.1038/nn.3092

[hbm24941-bib-0056] Winkler, A. M. , Ridgway, G. R. , Webster, M. A. , Smith, S. M. , & Nichols, T. E. (2014). Permutation inference for the general linear model. NeuroImage, 92, 381–397. 10.1016/j.neuroimage.2014.01.060 24530839 PMC4010955

[hbm24941-bib-0057] Zilverstand, A. , Huang, A. S. , Alia‐Klein, N. , & Goldstein, R. Z. (2018). Neuroimaging impaired response inhibition and salience attribution in human drug addiction: A systematic review. Neuron, 98, 886–903. 10.1016/j.neuron.2018.03.048 29879391 PMC5995133

