# Supplementary material for: Network failures: When incentives trigger impulsive responses
Source: Hum Brain Mapp. 2020 Mar 9;41(8):2216–28. doi: 10.1002/hbm.24941 (PMC7267965; doi:10.1002/hbm.24941)

**Supplementary Information**

**Methods:**

*Timeseries extraction.* Following the mass univariate GLM analysis, we investigated BOLD timeseries from several regions of interest for a) their ability to predict trial type and b) the causal role they play as part of a Dynamic Causal Model network.

To maximize power and sensitivity in the BOLD timeseries, we used individual subjects’ peaks within significant group mean activation in the premature>correct contrast. The ROIs were located within significant group mean activations and anatomically constrained to facilitate the interpretation of the results. In practice, the mean group activations were masked with anatomical ROIs from the Harvard Oxford atlas: IFC (pars opercularis), dorsal striatum (dStriatum), anterior cingulate (ACC) and parietal operculum. Subject-specific peak was not constrained to either side when group effects were bilateral (e.g. in the IFC for HC and SIB in money incentive context or SDI in drug incentive context).

In order to validate the relatively sparse GLM design, we selected several ROIs (including the ACC, IFC and dorsal striatum) that were associated with the premature>correct contrast. Next, we extracted and pre-processed the timeseries from those ROIs and extracted the activations for correct and premature responses (areas underneath the ROI timeseries highlighted in red and blue). These activations were then used to predict the response type in a logistic regression with subject-level random effects (see main Methods for additional details). This validation analysis revealed a strong discriminative effect of the BOLD activation in the selected ROIs on response type (Figure 1D and Supplementary Figure 3).

The validation analysis also included the parietal operculum when appropriate (for SDI and HC in the money incentive context and SDI in drug incentive context). DCMs used a subset of ROIs from this analysis, which were shown to be predictive of trial type, lending further credibility to the neurobiological data constraining the dynamic causal models.

Firstly, search space ROIs were created by taking the Harvard-Oxford anatomical atlas masks for bilateral inferior frontal cortex (IFC, pars opercularis), striatum (bilateral caudate, nucleus accumbens and putamen), anterior cingulate cortex (ACC) and parietal operculum regions. The anatomical ROI was masked by the group mean activations in the HC, SDI and SIB groups for money and drug incentive conditions, respectively, resulting in distinct search space ROIs for each group, which were then used to extract individual timeseries. Individual contrast parameter estimate maps (premature > correct_drug_for SDIs; premature > correct_money_for HCs and SIBs) were masked with the search space ROI, and FSL cluster (<https://fsl.fmrib.ox.ac.uk/fsl/fslwiki/Cluster>) was used to extract the coordinates of the maximum intensity voxel in the largest cluster for each ROI (IFC, Caudate, ACC, pO). A sphere of 5 mm radius was placed at the maximum intensity voxel and the first Eigenvariate from each ROI was extracted sing fslmeants (<https://fsl.fmrib.ox.ac.uk/fsl/fslwiki/Fslutils>).

The extracted timeseries were preprocessed using the rsHRF toolbox for SPM (Wu *et al*, 2013; Wu and Marinazzo, 2016) by deconvolving the timeseries with a canonical HRF with time and dispersion derivatives (TR=2s, length of HRF=32s), bandpass filtering (0.001-0.3Hz) and despiking the timeseries. Subsequently, the timeseries were upsampled by a factor of 100 using spline interpolation (interp1, MATLAB) and the total area for each of the anticipation windows (premature or correct) for the relevant money and drug conditions, were extracted using numerical integration (trapz, MATLAB, Appendix Figure S1, supplementary figure 1).

*Driving inputs for the DCM.* The driving inputs for the DCM assume that all trials activate the PFC areas including the ACC and IFC. This assumption is justified by other studies employing DCM to investigate stopping impulsivity (Rae *et al*, 2015, 2016), based on the expectation that impulse control requires top-down inhibition provided by the IFC and the ACC. Furthermore, data-driven experiments show this assumption to be justified in context of response inhibition in the Go/No-Go task (Ma *et al*, 2015), as DCM discovery studies show that ACC, vlPFC, dlPFC and hippocampus are reliable driving inputs for all Go/No-Go trials, whereas caudate has only been a reliable input for easy Go/No-Go trials.

Analyses results from all reported random effects DCMs (RFX) in the main text were verified and confirmed using fixed effects analyses (FFX).

**Results**

Inspection of the unaffected sibling group (SIB) revealed several points. Firstly, the SIB winning DCM family was also fully interactive in terms of fixed connections (Family exceedance probability$\approx$1). However, the location of task modulation varied substantially across participants (Figure S3). Models placing the modulation location at the ACC, IFC, or the connections between IFC and dStriatum or between the ACC and dStriatum all received some evidence. Bayesian Model Averaging revealed strong effective connectivity between the ROIs in the SIB group, but no significant task modulation inputs were identified due to interindividual variability. Since SIB also showed worse fit of the DCMs to data compared to the other groups, we focused on the more robust DCMs in HC and SDI.

Additionally, we noted that on average SIBS showed lower self-reported money valuations (Table 1). Taken together, attributing lower incentive salience to monetary rewards within the context of this study compared to the other two groups could have been critical in a) eliciting attenuated network activations and b) inter-individual variability in DCM task modulation in the SIB group.

**Main effect of condition on target stimulus duration**

The target stimulus duration was titrated to achieve 66% accuracy in responding, therefore providing an additional measure of task performance. As shown in Supplementary Figure 5, we report no main effect of the group (HC, SIB, SDI) on mean target duration (F_2, 129_=1.074, p=0.34). There were no interactive effects of group and condition F_2, 129_=1.534, p=0.22), although a very small but significant effect of condition was found F_1, 129_=4.07, p=0.045).

**References**

Ma L, Steinberg JL, Cunningham KA, Lane SD, Bjork JM, Neelakantan H, *et al* (2015). Inhibitory behavioral control: A stochastic dynamic causal modeling study comparing cocaine dependent subjects and controls. *NeuroImage Clin* **7**: 837–847.

Rae CL, Hughes LE, Anderson MC, Rowe JB (2015). The prefrontal cortex achieves inhibitory control by facilitating subcortical motor pathway connectivity. *J Neurosci* **35**: 786–94.

Rae CL, Nombela C, Rodríguez PV, Ye Z, Hughes LE, Jones PS, *et al* (2016). Atomoxetine restores the response inhibition network in Parkinson’s disease. *Brain* **139**: 2235–2248.

Wu GR, Liao W, Stramaglia S, Ding JR, Chen H, Marinazzo D (2013). A blind deconvolution approach to recover effective connectivity brain networks from resting state fMRI data. *Med Image Anal* **17**: 365–374.

Wu GR, Marinazzo D (2016). Sensitivity of the resting-state haemodynamic response function estimation to autonomic nervous system fluctuations. *Philos Trans R Soc A Math Phys Eng Sci* **374**: .

**Supplementary Table 1.** Whole brain group comparisons for the Premature > Correct response contrast in the drug incentive context.

| **SDI>HC group comparison** |  |  |  |  |  |  |
| --- | --- | --- | --- | --- | --- | --- |
| **Region (Brodmann Area)** | **Size** | **X_mm_** | **Y_mm_** | **Z_mm_** | **Z** | **P_FWE-cor_** |
| Primary Motor Cortex (BA6) - Left | 9343 | -52 | 2 | 46 | 4.23 | 6.96E-30 |
| Primary Motor Cortex (BA6) - Right | 1288 | 62 | -10 | 38 | 4.13 | 4.77E-07 |
| Temporal Pole (BA38) - Right | 787 | 40 | 20 | -32 | 4.44 | 0.000103 |
| Angular gyrus (BA39) - Right | 761 | 46 | -50 | 22 | 4.06 | 0.000139 |
| Amygdala - Left | 715 | -20 | -8 | -12 | 3.85 | 0.000241 |
| PCC (BA23) - Right | 591 | 10 | -54 | 8 | 3.31 | 0.00113 |
| PCC (BA31) - Left | 573 | -14 | -44 | 36 | 3.65 | 0.00142 |
| Occipital cortex (BA19) - Right | 362 | 30 | -82 | -12 | 3.97 | 0.0266 |
| OFC/vmPFC (BA11) - Left | 353 | -10 | 34 | -14 | 3.25 | 0.0305 |
| OFC (BA47) - Right | 353 | 32 | 38 | -12 | 4.02 | 0.0305 |
| OFC (BA47) - Left | 333 | -32 | 20 | -22 | 3.94 | 0.0412 |
| Thalamus (BA50) - Right | 338 | 20 | -28 | 8 | 3.6 | 0.0382 |
|  |  |  |  |  |  |  |
| Striatum* | 171 |  |  |  |  | 0.0365 |
| WM between Cau and Put |  | -22 | 2 | 18 | 3.31 | 0.0365 |
| Left Cau |  | -12 | 10 | 16 | 3.23 | 0.0365 |
| Left Cau |  | -14 | 8 | 20 | 3.15 | 0.0365 |
| Left anterior Cau |  | -14 | 18 | 12 | 3.12 | 0.0365 |
| WM between Put and Insula |  | -32 | -4 | 12 | 2.8 | 0.0365 |
| Left putamen |  | -22 | 2 | 8 | 2.49 | 0.0365 |
|  |  |  |  |  |  |  |
| **SDI>SIB group comparison** |  |  |  |  |  |  |
| **Region (Brodmann Area)** | **Size** | **X_mm_** | **Y_mm_** | **Z_mm_** | **Z** | **P_FWE-cor_** |
| Primary Motor Cortex (BA6) - Left | 23758 | -52 | 4 | 46 | 5.1 | <0.0001 |
| Occipital (BA19) - Left |  | -34 | -82 | 16 | 4.66 | <0.0001 |
| Occipital (BA18) – Left |  | -32 | -92 | 10 | 4.29 | <0.0001 |
| Superior temporal gyrus (BA22) – Left |  | -50 | -6 | -14 | 4.33 | <0.0001 |
| Thalamus (BA50) - Left |  | -10 | -20 | 12 | 4.31 | <0.0001 |
| Supramarginal gyrus (BA40) - Right | 1046 | 56 | -44 | 46 | 4.09 | 5.78E-06 |
| IFC (pars opercularis) (BA44) - Right | 646 | 52 | 14 | 32 | 3.8 | 0.000562 |
| Primary Motor Cortex (BA6) - Right | 570 | 46 | -14 | 40 | 3.72 | 0.00148 |
| Temporal lobe (BA21) - Right | 558 | 62 | -26 | -12 | 3.48 | 0.00173 |
|  |  |  |  |  |  |  |
| Striatum* | 350 |  |  |  |  | 0.00176 |
| WM between Put and Thal |  | -24 | -26 | 6 | 3.94 | 0.00176 |
| Left Putamen |  | -18 | -6 | 6 | 3.5 | 0.00176 |
| WM between Put and Insula |  | -36 | -2 | 2 | 3.41 | 0.00176 |
| Left dorsal caudate |  | -20 | -4 | 24 | 3.01 | 0.00176 |
| Left dorsal putamen |  | -24 | -4 | 14 | 2.86 | 0.00176 |
| WM between Put and Insula |  | -32 | -24 | 10 | 2.79 | 0.00176 |

*Striatum clusters were extracted from a 2nd level analysis with a whole striatum mask as shown in Figure 2 rather than the whole brain mask

WM, white matter; PCC, posterior cingulate cortex; OFC, orbitofrontal cortex; Cau, Caudate; Put, Putamen; Thal, thalamus; FWE, family wise error.

**Supplementary Table 2.** Results from the hierarchical logistic regression analyses with ROI activation as predictors of premature or correct trials.

| Healthy Controls (money context) summary | | | | | |
| --- | --- | --- | --- | --- | --- |
|  | Estimate | SE | Z-value | Pr(>\|z\|) |  |
| Intercept | -2.294 | 0.164 | -14.0 | 2E-16 | *** |
| IFG | 0.005 | 0.005 | 0.9 | 0.352 |  |
| ACC | 0.018 | 0.005 | 3.3 | 0.001 | *** |
| CAU | 0.009 | 0.004 | 2.2 | 0.030 | * |
| PO | 0.007 | 0.005 | 1.4 | 0.149 |  |
|  |  |  | BIC | 1379.5 |  |
|  |  |  |  |  |  |
| Healthy Controls (drug context) summary | | | | | |
|  | Estimate | SE | Z-value | Pr(>\|z\|) |  |
| Intercept | -2.294 | 0.164 | -14.0 | 2E-16 |  |
| ACC | 0.014 | 0.003 | 4.4 | 1E-05 | *** |
|  |  |  | BIC | 1320.6 | *** |
|  |  |  |  |  |  |
| Unaffected Siblings (money context) summary | | | | | |
|  | Estimate | SE | Z-value | Pr(>\|z\|) |  |
| Intercept | -2.169 | 0.125 | -17.3 | 2E-16 | *** |
| IFG | 0.010 | 0.003 | 3.1 | 0.002 | ** |
| ACC | 0.014 | 0.003 | 4.0 | 7E-05 | *** |
| CAU | 0.001 | 0.003 | 0.3 | 0.77111 |  |
|  |  |  | BIC | 1418.8 |  |
|  |  |  |  |  |  |
| SDIs (money context) summary | | |  |  |  |
|  | Estimate | SE | Z-value | Pr(>\|z\|) |  |
| Intercept | -1.916 | 0.135 | -14.2 | 2E-16 | ******* |
| IFG | 0.029 | 0.005 | 6.1 | 1E-09 | ******* |
| ACC | -0.015 | 0.005 | -2.9 | 0.004 | ****** |
| PO Left | 0.003 | 0.005 | 0.6 | 0.574 |  |
| PO Right | 0.013 | 0.005 | 2.5 | 0.011 | ***** |
|  |  |  | BIC | 1503.1 |  |
|  |  |  |  |  |  |
| Stimulant Dependent Individuals (drug context) summary | | | | | |
|  | Estimate | SE | Z-value | Pr(>\|z\|) |  |
| Intercept | -1.830 | 0.111 | -16.4 | 2E-16 | ******* |
| IFG | 0.008 | 0.004 | 1.9 | 0.064 |  |
| ACC | 0.000 | 0.005 | -0.04 | 0.971 |  |
| CAU | 0.014 | 0.005 | 3.0 | 0.003 | ****** |
| PO | 0.008 | 0.005 | 1.8 | 0.077 |  |
|  |  |  | BIC | 1553.4 |  |

IFG, inferior frontal gyrus; ACC, anterior cingulate cortex; Cau, caudate; PO, parietal operculum; SE, standard error; BIC, Bayesian Information Criterion.

**Supplementary Figure 1**. Example timeseries from a single participant used in the validation analysis of the fMRI GLM, with correct and premature trials modelled as events of interest.


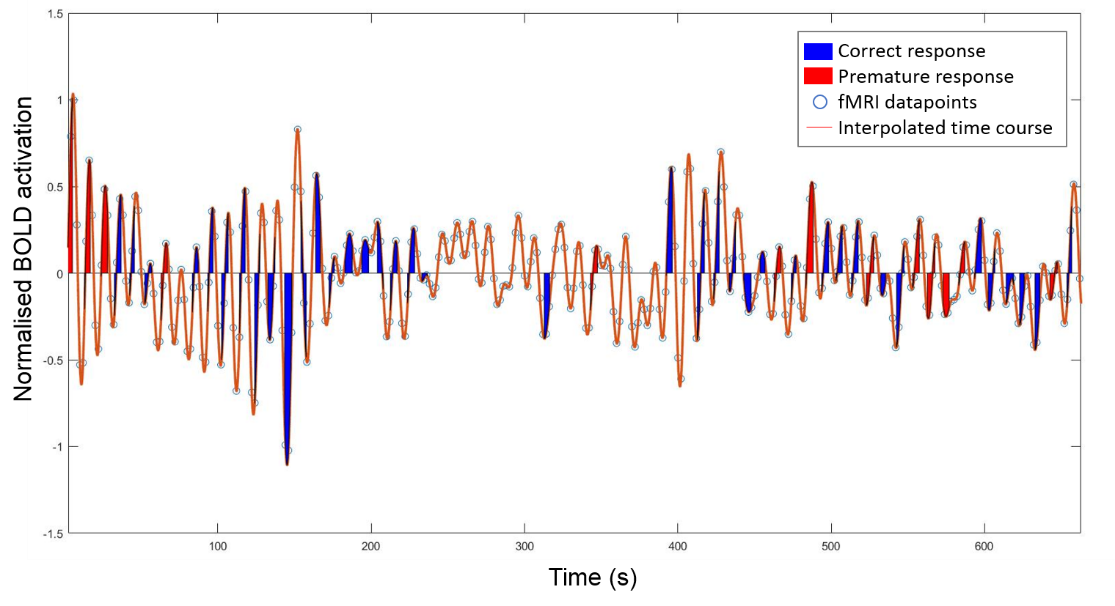


**Supplementary Figure 2**. Two-way interactions between group and incentive (money versus drug) in the striatum ROI. Differential response to incentives for SDI versus HC are shown in red/orange and for SDI versus SIB in blue. A significant interaction between group (HC vs SDI, SIB vs SDI) and incentive was found in the left caudate and left putamen. Z=[0 -2 4 8 10 14 16 18],  (Multiple comparison corrected using FSL randomise and thresholded at p<0.05 FWE).


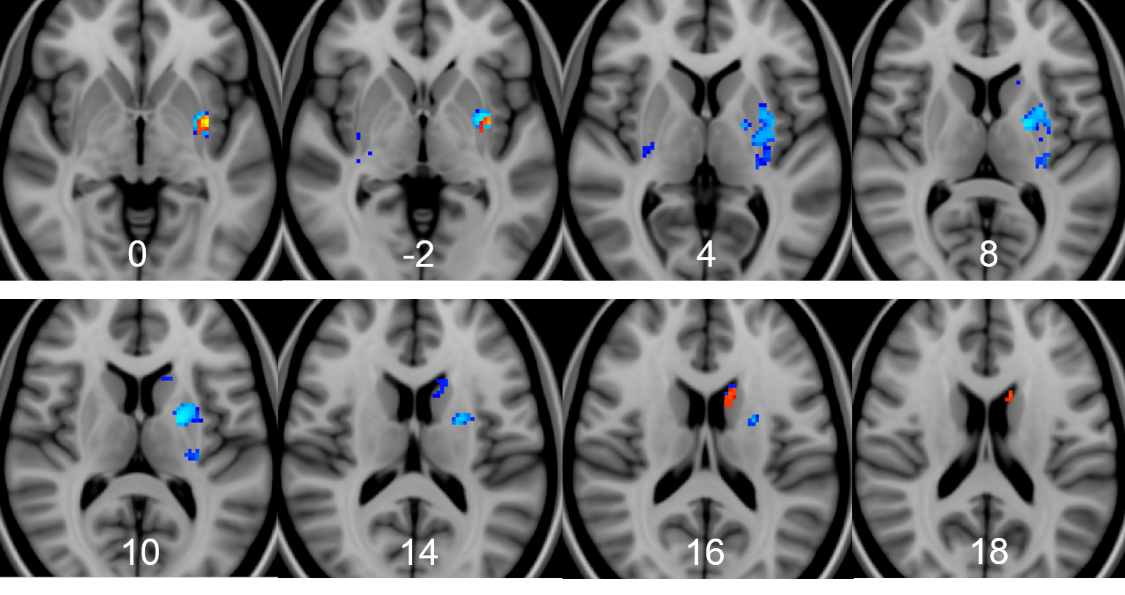


**Supplementary Figure 3.** ROI placements for each subject and their average time courses with 95% confidence intervals for premature and correct trial types. The first column shows inferior frontal cortex (IFC), dorsal anterior cingulate (dACC) and dorsal striatum (dSTR) spheres from each participant overlaid on the MNI template to illustrate the locations of the ROIs. Individual spheres for SDIs are shown in red and HC spheres are shown in blue. Second column shows average brain activation time courses obtained from deconvolved, denoised and interpolated BOLD signals for HCs in money condition, whereas the third and fourth columns shows average brain activation for each ROI in the SDIs in the money and drug conditions, respectively. The bold portion of the X-axis highlights the anticipation phase during which the premature response was made (premature trials in red), or which was immediately followed by an on-target response (correct trials in black). Anticipation phase duration varied randomly in duration between 3.5 and 5s. Note that these ROIs were used for validation analysis in Figure 1D and DCM analysis in Figure 4. While average SDI drug incentive time courses show overlap between premature and correct trials, this is likely due to high interindividual variability and did not significantly reduce within-subject prediction of trial type from ROI activation levels.


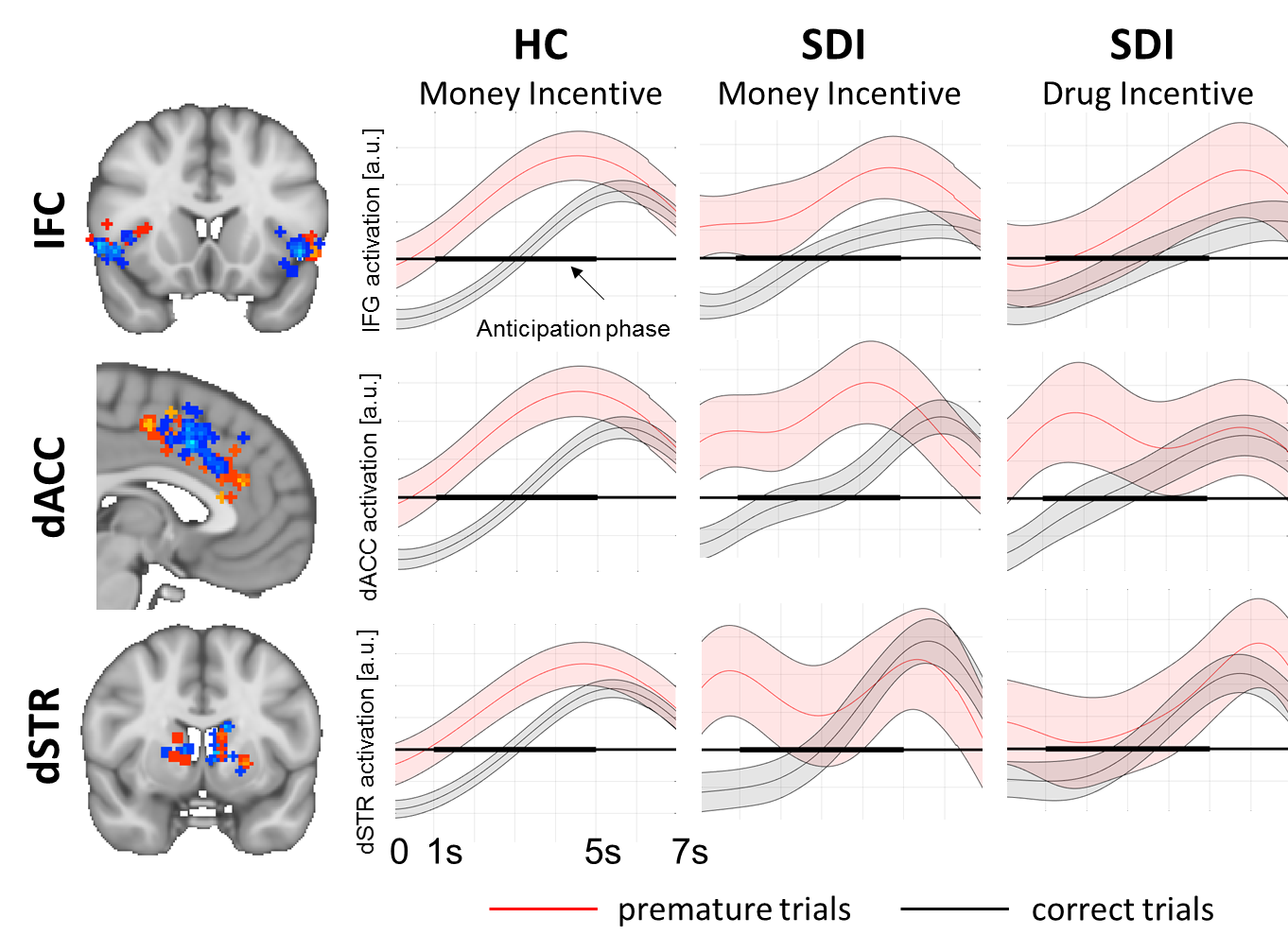


**Supplementary Figure 4.** DCM results including the SIB group. (A) Similarly to the findings from the SDI and HC groups, the fully interactive model reliably characterises the network connectivity in the unaffected siblings (SIB). (B) In contrast to the SDI and HC, the SIB group showed substantial interindividual variability, mirrored by the lack of a consistently winning model within the winning model family. (C) This was accompanied by an absence of significant modulatory effects as the location of task modulation varied across individuals. (D) Depiction of all models included in the BMS and BMA analyses. The first row contains models 1-3 (left to right), while the second and third rows contain models 4-6 and 7-9, respectively.


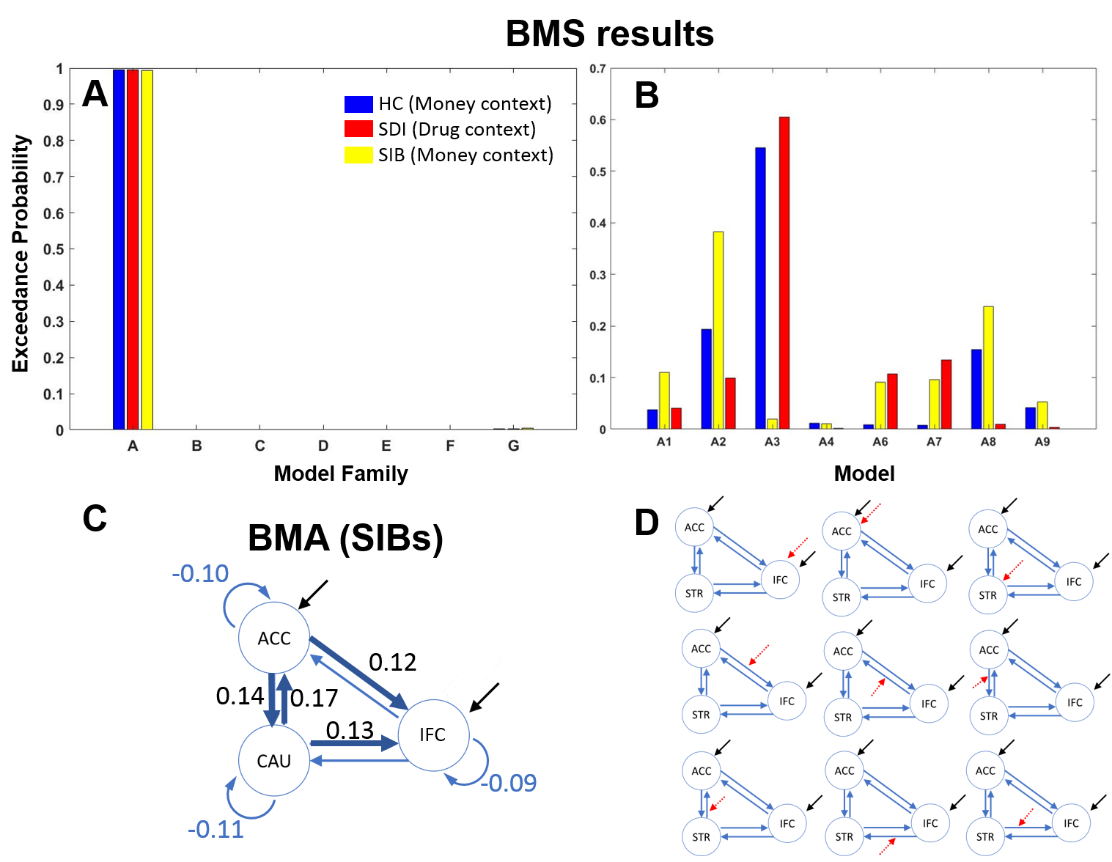


**Supplementary Figure 5.** Mean target stimulus duration in the HC, SIB and SDI in the drug and money conditions (A) and mean target duration difference between the conditions (B).


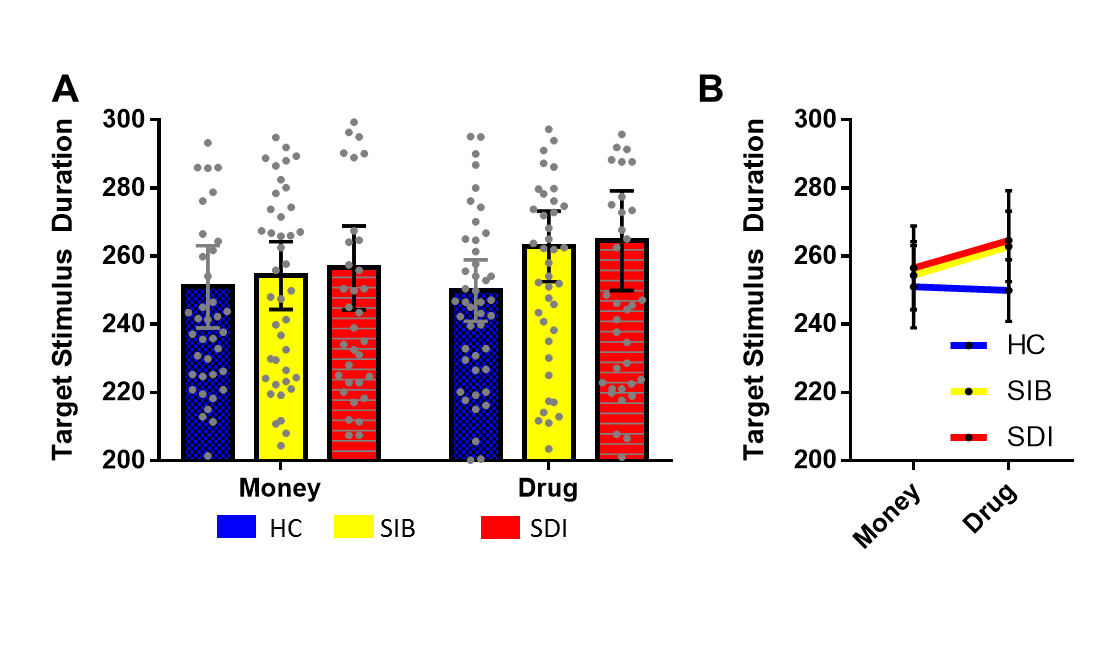

Supplement: Supplementary file 1 — Appendix S1: Supporting information [file HBM-41-2216-s001.docx]
